# Learning to simulate realistic human diffuse reflectance spectra

**DOI:** 10.1117/1.JBO.31.2.026004

**Published:** 2026-02-26

**Authors:** Marco Hübner, Ahmad Bin Qasim, Alexander Studier-Fischer, Maike Rees, Viet Tran Ba, Jan-Hinrich Nölke, Silvia Seidlitz, Jan Sellner, Janne Heinecke, Jule Brandt, Berkin Özdemir, Kris Dreher, Alexander Seitel, Felix Nickel, Caelan Max Haney, Karl-Friedrich Kowalewski, Leonardo Ayala, Lena Maier-Hein

**Affiliations:** a German Cancer Research Center (DKFZ), Division of Intelligent Medical Systems, Heidelberg, Germany; b Heidelberg University, Faculty of Mathematics and Computer Science, Heidelberg, Germany; c National Center for Tumor Diseases (NCT) Heidelberg, a partnership between German Cancer Research Center and Heidelberg University Hospital, Heidelberg, Germany; d Helmholtz Information and Data Science School for Health, Karlsruhe/Heidelberg, Germany; e Heidelberg University, Department of General, Visceral, and Transplantation Surgery, Heidelberg, Germany; f Heidelberg University, Medical Faculty, Heidelberg, Germany; g University Medical Center Mannheim, Medical Faculty of the University of Heidelberg, Department of Urology and Urosurgery, Mannheim, Germany; h German Cancer Research Center (DKFZ), Division of Intelligent Systems and Robotics in Urology (ISRU), Heidelberg, Germany; i DKFZ Hector Cancer Institute at the University Medical Center Mannheim, Mannheim, Germany; j Heidelberg University, Department of Physics and Astronomy, Heidelberg, Germany; k University Medical Center, Hamburg-Eppendorf, Department of General, Visceral, and Thoracic Surgery, Hamburg, Germany; l University Hospital Leipzig, Department of Urology, Leipzig, Germany; m Heidelberg University Hospital, Surgical Clinic, Surgical AI Research Group, Heidelberg, Germany

**Keywords:** hyperspectral imaging, Monte Carlo simulation, surrogate model, diffuse reflectance, tissue model, neural scaling

## Abstract

**Significance:**

Hyperspectral imaging is a noninvasive, cost-effective modality with transformative clinical potential. Its adoption is limited by the lack of accurate and efficient methods that relate spectra to tissue parameters, essential for both AI training and validation of imaging methods, as gold standard Monte Carlo (MC) simulations remain prohibitively computationally expensive.

**Aim:**

We aim to develop a scalable and accurate method for generating realistic tissue reflectance spectra in support of AI development and validation in biomedical imaging.

**Approach:**

We trained a general-purpose neural surrogate model on >50 million MC simulations based on a flexible multilayer tissue model. We validated our model against >5000 open surgery *in vivo* hyperspectral images, annotated with 23 tissue classes for stratified performance analysis. In addition, we qualitatively evaluated clinical potential by testing whether surrogate-generated spectra enable recovery of organ-specific oxygenation dynamics in a controlled porcine aortic clamping experiment.

**Results:**

The surrogate model achieved accuracy matching MC simulations with 5–10 million photons while delivering inference five orders of magnitude faster. Across 140 million human tissue spectra, it improved spectral recall by 13–48 percentage points over existing surrogate models. Scaling analyses revealed a power law relationship between training dataset size and test error, enabling the prediction of training data requirements for target accuracy. Our porcine study suggests that the synthetic data generated with the surrogate model is suitable for recovering organ-specific $s_{t}O_{2}$ trajectories.

**Conclusion:**

Neural surrogate models can achieve MC-level accuracy and *in vivo* realism at negligible inference cost, enabling large-scale, compute-efficient data generation for biomedical optics and robust AI development for clinical applications.

## 1 Introduction

Hyperspectral imaging (HSI) is a promising imaging modality for the noninvasive characterization of tissue properties during surgery,^1^^–^^4^ enabling quantitative hemodynamic assessment for various in-human applications, including monitoring cerebral oxygenation, diagnosis of breast cancer, and real-time detection of ischemia or renal malperfusion.^5^^–^^9^ Despite these capabilities, most *in vivo* studies have involved only small cohorts of patients, leaving much of HSI’s clinical potential unexplored. Progress has further been slowed by the absence of effective methods to quantify absolute tissue parameters, which face validation challenges due to the lack of pixel-wise ground-truth measurement *in vivo*. Consequently, most HSI-based methods have been validated only on relative changes in superficial skin oxygenation,^10^^–^^13^ prohibiting their intraoperative use, particularly for detecting local hypoxia, hyperoxia, and ischemia. Robust quantification could open new avenues for perioperative spectral imaging and diagnosis of a broad range of diseases.^14^^,^^15^

Supervised deep learning methods have shown promise for addressing the quantification problem^7^^,^^8^^,^^16^ but depend on accurate ground-truth mappings between spectral measurements and tissue parameters, which remain a major bottleneck. Although Monte Carlo (MC) simulations, the gold standard for modeling light transport, offer high accuracy, they are computationally expensive, limiting their feasibility for real-time use or large-scale dataset generation.^11^^,^^13^^,^^17^^,^^18^

To accelerate computation, researchers have explored alternatives such as the diffusion approximation,^19^^–^^21^ semi-analytical models based on Beer-Lambert or Kubelka-Munk theory,^22^^–^^24^ and the adding-doubling method.^18^^,^^25^^,^^26^ More recently, neural network-based surrogate models have been proposed to emulate MC outputs^13^^,^^27^^–^^30^ with an acceleration of inference by a factor of 1000 to 40,000.^28^^,^^30^

However, the realism of surrogate models not only depends on the light transport simulation but also on the parametrization and variability of the input tissue representations. Existing state-of-the-art (SOTA) surrogate models have often targeted specific tissue types, with constrained parameter ranges and static configurations,^13^^,^^28^^,^^29^^,^^31^ or adopted generic but simplified, single-layer tissue models.^22^^,^^24^^,^^30^ These restrictions reduce realism and limit applicability to heterogeneous, clinically relevant scenarios: It has been shown that single-layer models underestimate chromophore concentrations such as hemoglobin and melanin when used to address the quantification problem^32^ and have failed to capture spatial reflectance variations across source-detector separations.^10^ Moreover, validation, similar to methods tackling the quantification problem, has been limited to small datasets from healthy volunteers with narrow anatomical or pathological diversity,^8^^,^^10^^,^^12^^,^^17^^,^^21^^,^^28^ leaving unclear how well existing tissue and surrogate models capture the spectral variability encountered in surgical settings.

**Fig. 1:**
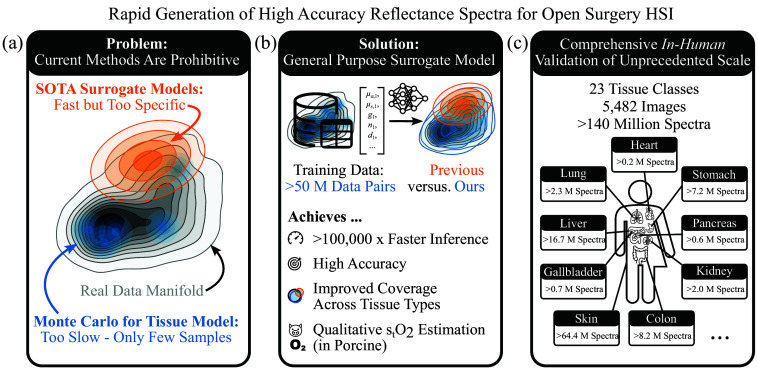
Core contributions. (a) SOTA reflectance simulators are limited by high computational cost or incomplete data coverage. (b) We propose a general-purpose surrogate model capable of generating diverse human tissue reflectance spectra with Monte Carlo-level accuracy while delivering orders-of-magnitude faster inference and capable of $s_{t}O_{2}$ estimation *in vivo*. (c) Validation was conducted on a dataset of unprecedented size, comprising over 5000 *in vivo* human hyperspectral images annotated with 23 tissue classes.

To address these challenges, we make the following contributions (Fig. 1):

• We develop a multilayer general-purpose surrogate model capable of realistically generating human tissue reflectance spectra with MC-level accuracy and five orders of magnitude faster inference.
• We conduct scaling experiments revealing power-law relationships between training dataset size and spectral fidelity of our surrogate model, offering predictive guidance for efficient training data simulation strategies to target specific reflectance accuracy thresholds.
• We validate both our tissue and surrogate model, in comparison to SOTA methods, using a large-scale *in vivo* surgical HSI dataset of over 5000 images and 140 million spectra across 23 tissue classes, representing the most extensive evaluation of its kind to date.
• We demonstrate that surrogate-generated reflectance spectra enable recovery of organ-specific $s_{t}O_{2}$ dynamics during ischemia and reperfusion in a porcine aortic-clamping experiment.

## 2 Materials and Methods

**Fig. 2:**
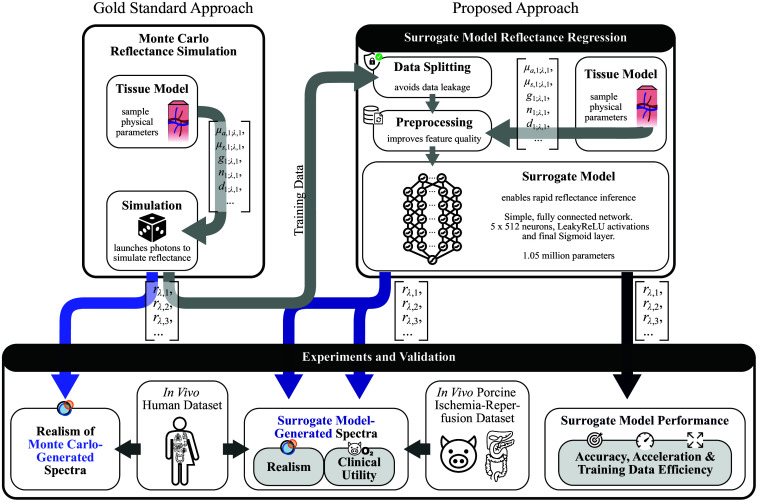
Learning to simulate reflectance spectra: model design and validation. For a (multilayer) tissue model, the reflectance $r_{\lambda }$ is first obtained from Monte Carlo (MC) simulations (Sec. 2.1). After training the neural network surrogate model on the MC simulations, it can generate diffuse reflectance spectra at negligible computational cost based on tissue model specifications (Sec. 2.2). Validation proceeds in three steps (Sec. 2.3): (i) surrogate model performance (ii) realism assessment of both MC- and surrogate-generated spectra with human *in vivo* data and (iii) qualitatively evaluating clinical potential in an arterial occlusion experiment.

This work aims to enable accurate, scalable synthesis of realistic tissue reflectance spectra for AI training and imaging system validation. We approach this task in three main steps:

1. Simulation pipeline design: We developed a simulation pipeline for SOTA tissue models (Fig. 2, left side, Sec. 2.1).
2. Surrogate model design: Based on the simulated data, we trained high-fidelity, general-purpose surrogate models to generate spectra orders of magnitude faster than MC simulations (Fig. 2, right side, Sec. 2.2).
3. Experimental design: We benchmarked the surrogate model against MC simulations (Fig. 2, bottom right, Sec. 2.3.1), assessed realism relative to SOTA alternatives on large-scale in vivo human data (Fig. 2, bottom left, Sec. 2.3.2), and performed an in *vivo* qualitative analysis to demonstrate the clinical potential of the synthetic spectra (Fig. 2, bottom middle, Sec. 2.3.3).

Together, these components form a surrogate modeling framework that combines the accuracy of MC simulations with the scalability of machine learning, enabling realistic, high-throughput spectral synthesis for biomedical optics.

### 2.1 Monte Carlo Reflectance Simulation and Tissue Models

**Fig. 3:**
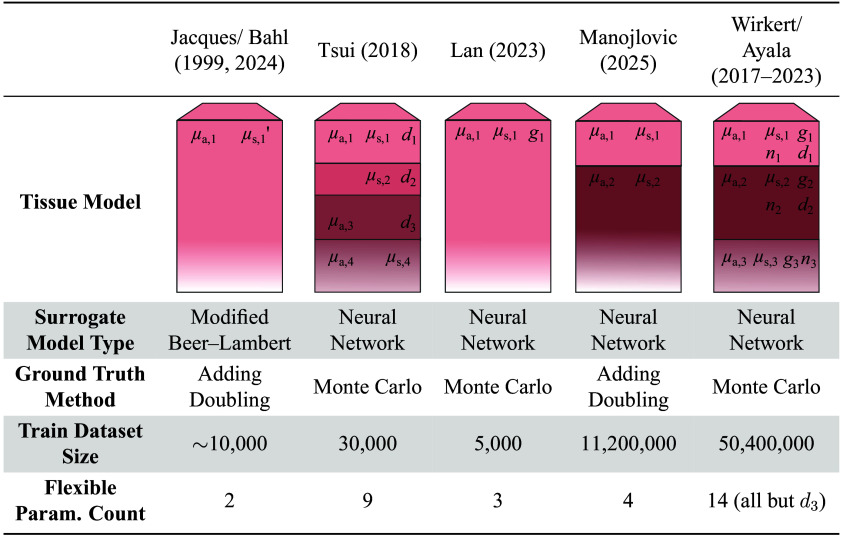
Our tissue model implementation offers the highest parameter flexibility and the largest training dataset. Only our model varies the refractive index n, whereas anisotropy g and thickness d have also often been fixed in prior works. “Ground Truth Method” denotes the data simulation approach, “Train Dataset Size” is the number of simulated physical parameter sets, and “Flexible Parameter Count” lists independently varied physical parameters.

As a baseline for tissue model realism and to train and evaluate the surrogate models of Fig. 3, we reimplemented a diverse set of homogeneous multilayer and single-layer models, including a three-layer epithelial design previously used in the work of our group, as well as several reimplemented SOTA models from prior literature for comparison. All models were simulated with a standardized GPU-accelerated multilayer Monte Carlo pipeline,^33^ adapted from GPUMCML.^34^^,^^35^ The setup used a pencil-beam light source, the Henyey-Greenstein phase function for scattering anisotropy, and infinitely wide layers for all simulations to ensure reproducibility and fair comparison across implementations. Dataset-specific ranges and photon counts are detailed in the individual experiment descriptions and in Secs. S1–S3 in the Supplementary Material.

#### Wirkert and Ayala et al.^7^^,^^16^^,^^36^ (ours)

Our work uses the three-layer epithelial tissue model introduced by Wirkert and Ayala et al.^7^^,^^16^^,^^36^ which extended typical multilayer designs by varying absorption, scattering, anisotropy, and refractive index, providing full parameter flexibility for more nuanced light-tissue interaction modeling. Absorption was modeled with hemoglobin as the sole chromophore at a constant tissue oxygenation ($s_{t}O_{2}$) across layers, determined by a common arterial oxygenation ($s_{a}O_{2}$). Computation of the absorption coefficient $\mu_{a}$ and scattering coefficient $\mu_{s}$ followed Ayala et al.^7^ To avoid artifacts from photon escape at the base, the bottom layer thickness was fixed at 20 cm, approximating a semi-infinite medium.

#### Jacques^22^ and Bahl et al.^24^

The tissue model adopted by Bahl et al.^24^ used a single-layer design with hemoglobin as the sole absorber and a globally fixed refractive index n of 1.33, 1.35, or 1.44 per dataset.

#### Tsui et al.^28^

The skin-specific four-layer tissue model assigned a fixed anisotropy value to each layer and used a single global refractive index. Absorption and scattering coefficients were often linked across layers, constraining parameter variation.^28^

#### Lan et al.^30^

A general-purpose, single-layer design was adopted and absorption, scattering, and anisotropy were varied independently. The original work did not specify the refractive index, which we thus fixed to n=1.35.

#### Manojlovic et al.^13^

The two-layer skin model incorporated multiple chromophores, including melanin, hemoglobin, bilirubin, and cytochrome c oxidase but fixed scattering power $b_{\mathrm{Mie}}$, fixed wavelength-dependent anisotropy g(λ), fixed wavelength-dependent refractive index n(λ), and fixed layer thickness d globally.

The semi-analytical surrogate model of Yudovsky et al.^23^ was excluded from evaluation due to inconsistencies between its original publication, erratum, and subsequent reimplementations.^23^^,^^24^^,^^37^

### 2.2 Surrogate Model Reflectance Regression

To address the prohibitive computational cost of MC simulations, we developed a general-purpose neural network surrogate model that regresses diffuse reflectance spectra from physical tissue parameters.

#### 2.2.1 Surrogate model architecture

Figure 2 shows the general architecture of the surrogate model. The five physical parameters per tissue layer and wavelength shown in Fig. 3 served as neural network input, generating diffuse reflectance values $r_{\lambda }$ as output. Physiological parameters were first converted to physical parameters using previously published mappings.^7^^,^^13^^,^^24^^,^^28^

The surrogate model, implemented using PyTorch Lightning,^38^^,^^39^ comprised five fully connected hidden layers with 512 neurons each, together with an input and output layer totaling ∼1.05 million trainable parameters. The hidden layers used Leaky ReLU activation functions with a slope of 0.2, whereas the output layer employed a sigmoid activation to map predictions to the diffuse reflectance range. Network weights were initialized using Kaiming initialization with a normal distribution,^38^^,^^40^ and biases were set to zero. No regularization techniques, such as dropout or batch normalization, were applied, as no signs of overfitting were observed during training.

#### 2.2.2 Surrogate model training

##### Data splits

The datasets were divided into training, validation, and test splits comprising 70%, 10%, and 20% of the data, respectively. To prevent data leakage across wavelengths, the split for physiologically sampled datasets was performed at the spectrum level. All reported performance metrics and visual evaluations were conducted solely on the respective test data splits after finishing model development.

##### Preprocessing

The preprocessing pipeline involved two transformation steps to improve feature quality: absorption and scattering coefficients, as well as layer thicknesses, were log-transformed (decadic) to reduce their wide dynamic ranges, before standardizing all parameters using z-score normalization. The normalization parameters were computed exclusively on the training (k-fold) split and stored for consistent application during inference, preventing any data leakage from validation or test splits. Reflectance values were left unprocessed due to their naturally bounded range between 0 and 1, which already provided well-distributed values suitable for neural network training.

##### Training protocol

Our training protocol balanced convergence speed and training stability, using Sophia,^41^ an approximate second-order optimizer, which demonstrated faster convergence compared with Adam^42^ in our preliminary experiments. The learning rate was set to $10^{-4}$ with a weight decay of 0.001 to prevent overfitting. For reproducing related work, the optimizer scale update parameter ρ was increased to 0.1. Hyperparameters were explored and optimized with Optuna^43^ to evaluate gains from nondefault, complex configurations, but no substantial improvements were observed during extensive hyperparameter exploration. Performance depended mainly on learning rate and batch size, so the simple, stable configuration previously described was retained for all experiments.

We used PyTorch learning rate scheduling,^38^ halving the learning rate on plateaus of the validation loss until reaching the minimum learning rate of $10^{-8}$. The patience of the learning rate scheduler, typically 20 epochs, was adapted to the training duration and increased for smaller datasets that required more epochs to converge. The number of training epochs was set to 20–50 times the patience, with a minimum of 500 epochs. Both larger and smaller datasets required more iterations for convergence, and the batch size was increased for the largest datasets to improve training stability. Training used 32-bit precision, as 16-bit degraded performance, and no data augmentation (e.g., noise addition) was applied, given the lack of observed improvements in preliminary experiments.

The performance of our reimplemented models is compared with the reported performance from original publications in Sec. S5 and Tables S2 and S3 of the Supplementary Material.

### 2.3 Experimental Design

Our study was designed to address the following three research questions (RQs) regarding the fidelity and utility of the proposed general-purpose surrogate model:

**(RQ1) Surrogate model performance:** How well do surrogate models match Monte Carlo accuracy, accelerate inference, and scale in performance with training dataset size?

**(RQ2) Model realism and utility:** How well do current physiological Monte Carlo tissue and surrogate models capture *in vivo* human reflectance data variability?

**(RQ3) Clinical use case:** To what extent do synthetic reflectance spectra support recovery of organ-specific $s_{t}O_{2}$ dynamics *in vivo*?

All simulated and surrogate-generated spectra were adapted to match the optical characteristics of the light source and camera in the *in vivo* benchmarking dataset, following Ayala et al.^7^ This spectral adaptation step is crucial for maintaining consistency between simulated and measured data, enabling meaningful reflectance spectra comparisons across different data sources. Our surrogate model also naturally supports post hoc adaptation to other camera systems or acquisition conditions by applying the corresponding spectral response functions, or adding. e.g., shot noise, without retraining.

#### 2.3.1 Surrogate model performance

We evaluated surrogate model performance (RQ1) with respect to regression fidelity, theoretical accuracy limits, inference acceleration, and data efficiency on the test split of our Surrogate Model Development Dataset. This multidimensional evaluation provides a comprehensive picture of the surrogate model’s capabilities and limitations.

##### Surrogate model development dataset

For fair comparison and faithful reimplementation, all surrogate models used the same neural network architecture; only the training datasets, their underlying tissue models, and the resulting input layer differed. Training employed fivefold cross-validation with a fixed global validation set. Datasets from related work followed their original evaluation protocols and were not split into folds. For the neural data scaling experiments, we added samples only to the training split while keeping validation and test sets fixed. In line with the original implementation, the dataset of Manojlovic et al.^13^ was halved before splitting into train, validation, and test splits.

Our primary development dataset comprised one million Latin hypercube-sampled physiological parameter sets at 15 wavelengths, prioritizing parameter diversity over spectral resolution. It was later expanded to five million sets for neural data scaling analyses. Baselines from prior work^13^^,^^22^^,^^24^^,^^28^^,^^30^ were resimulated using the published parameter ranges, photon budgets, and sampling schemes. Full specifications are provided in Sec. S2 and Table S1 of the Supplementary Material.

##### Regression fidelity

Goodness of fit was assessed using the mean absolute error (MAE) and mean absolute percentage error (MAPE) across all reflectance values on the test split of our Surrogate Model Development Dataset. To establish an estimate for the best attainable fit, we repeated the evaluation against MC simulations of the same parameters generated with ten times more photons, reducing simulation noise and obtaining a best-case estimate for both MAE and MAPE. To further contextualize surrogate model performance with simulation uncertainty, we derived theoretical MC error bounds by modeling photon transport as a Bernoulli process. In this simplified model, each photon is either absorbed or escapes at the bottom (failure, $N_{\text{escaped}}$) or is scattered and detected (success, $N_{\text{returned}}$). The reflectance corresponds to the success probability, with the maximum likelihood estimator

r^=NreturnedNtotal, (1)

where $N_{\text{total}}=N_{\text{returned}}+N_{\text{escaped}}$. Using Eq. (1), the binomial distribution B(Ntotal,r^) yields the standard deviation

σ^=Ntotal·r^·(1−r^) (2)

and the coefficient of variation (CoV), quantifying the relative MC error σ/μ

CoV=σμ≈σ^Ntotal·r^=1−r^Ntotal·r^, (3)

also used in Tsui et al.^28^ Equations (1)–(3) allow us to approximate absolute ($\hat{\sigma }$) and relative (CoV) lower error bounds from first principles using only observed reflectance values and the simulated photon count.

Surrogate model accuracy was assessed by visually comparing the distribution of absolute percentage errors (APEs) on the test split of our Surrogate Model Development Dataset against both theoretical and empirical reference values: The 95% prediction interval (PI) of the estimated binomial MC error distribution and the CoV for simulations with five and ten million photons as theoretical lower error bounds, and empirical APE computed against simulations with ten times more photons as empirical lower error bound.

##### Inference acceleration

To quantify the surrogate model’s computational speedup, we measured the time to generate 1000 spectra of 15 wavelengths with MC simulations across several photon counts chosen to match the surrogate’s accuracy, and compared these times with surrogate inference runtime. Benchmarks were run on a workstation with an NVIDIA RTX 3090 GPU and an AMD Ryzen 9 5900X CPU with 12 cores. The MC simulations used the same configuration as in our Surrogate Model Development Dataset. Each setting was repeated 30 times with randomly sampled, independent parameter sets to obtain robust estimates.

Surrogate inference runtime measurements included both physiological parameter preprocessing and neural network forward pass for the same parameter batch size, providing a fair end-to-end inference time comparison. For comprehensive performance analysis, a detailed runtime-batch size ablation study is provided in Fig. S1 in the Supplementary Material.

##### Data efficiency and neural data scaling

To analyze data requirements for optimal surrogate model performance, we investigated the data scaling behavior of the surrogate model using neural scaling laws:^44^^–^^46^ Empirical evidence across various domains has shown that model error, regardless of architecture, often follows a power-law decay^24^^,^^44^^–^^47^ with increasing compute, model, or dataset size. In line with these findings, we modeled the surrogate model test error as a function of training data volume $N_{\text{data}}$ such as

$$\text{Model Error}(N_{\text{data}})=a\cdot N_{\text{data}}^{b}+c,$$ (4)

where a denotes a scaling factor, b the respective power law exponent, and c the irreducible error that cannot be improved upon with more training data.

Training datasets were subsampled to 0.1%, 0.25%, 0.5%, 1%, 2.5%, 10%, 25%, 50%, and 100% of the full training dataset, with the smallest fractions (0.1% to 0.5%) chosen to approximate the dataset sizes of earlier tissue-model studies^28^ and to provide continuity between prior work and our full scaling analysis toward the empirical lower error bound. In addition, the same datasets have been generated with different numbers of photons varying between 100,000 and 100 million to investigate the trade-off between data quality and computational efficiency in dataset generation. To further probe scaling for models that have not converged within the given data budget, datasets have been expanded post hoc to up to 350% and 600%. For each training dataset size, the test MAE was computed on the same global held-out test split using the final model checkpoint. The power law of Eq. (4) was fit to the test MAE across training dataset sizes and folds with SciPy’s curve_fit method on log-scaled data, using the trust-region reflective algorithm and parameter bounds [0,−5,0] to $\left[\infty ,5,10^{-2}\right]$.^48^

We reused the two previous lower MC error bounds as reference, derived from the standard deviation of the Bernoulli-based MC error model and from the MAE between the test split of MC simulations with low(er) and very high photon count. Further scaling experiments of the reimplemented SOTA models were conducted in Fig. S8 in the Supplementary Material.

#### 2.3.2 Monte Carlo and surrogate model realism

To evaluate both the realism of the tissue model and the surrogate model (RQ2), we systematically compared the model spectra against the *In Vivo* Human Benchmarking Dataset stratified by tissue label class, evaluating how well they captured the variability and manifold structure of human tissue reflectance and thereby their clinical utility. To ensure a fair and rigorous comparison across all models, we reproduced relevant SOTA tissue and surrogate models, verifying that our reimplementations achieved comparable or better performance than reported in their original work (see Table S3 in the Supplementary Material). All datasets were subsampled to equal sizes, eliminating dataset size as a confounding factor: MC-based tissue models were evaluated with 70,000 spectra, limited by prior protocols, whereas surrogate models were assessed with 100,000 spectra, enabling direct performance comparison across different modeling paradigms.

##### *In vivo* human benchmarking dataset

To evaluate the realism of simulated spectra against clinical reference data, we employed a comprehensive *in vivo* hyperspectral dataset acquired during surgical procedures. The data collection was conducted with two TIVITA^®^ Tissue hyperspectral cameras (Diaspective Vision GmbH, Germany), each capturing 640×480  pixel images across 100 wavelength bands spanning 500 to 1000 nm with a spectral resolution of 5 nm.

The dataset comprised 145,884,282 pixel spectra extracted from 5482 images. These spectra represented 23 distinct annotated tissue and organ classes, encompassing abdominal and thoracic anatomy: stomach, small bowel, colon, liver, gallbladder, pancreas, kidney, spleen, bladder, diaphragm, esophagus, omentum, peritoneum, heart, lung, subcutaneous fat, visceral fat, hepatic ligament, artery, vein, muscle, skin, and cauterized tissue.

Data collection was conducted under the SPACE trial (SPectrAl Characterization of organs and tissuEs during surgery) at Heidelberg University Hospital. The study was approved by the Ethics Committee of the Medical Faculty of Heidelberg University (Approval ID: S-459/2020) and was conducted in accordance with the Declaration of Helsinki, Good Clinical Practice (GCP), and CONSORT reporting guidelines. Informed consent was obtained from all participants, and the trial was officially registered in the Research Registry on November 23, 2020 (ID: researchregistry6281).

##### Tissue model validation datasets

To assess realism and enable fair comparison, we generated three Monte Carlo datasets: A dataset based on our multilayer tissue model (Wirkert and Ayala et al.^7^^,^^16^^,^^36^) served to establish an *in vivo* data coverage baseline for later comparison with the surrogate model, whereas the two datasets reimplementing the multiwavelength physiological models of Jacques and Bahl et al.^22^^,^^24^ and Manojlovic et al.^13^ provided alternative tissue models used in previous work. Details on parameter ranges, sampling schemes, layer thicknesses, photon budgets, and wavelength grids are provided in Sec. S1 and Table S1 in the Supplementary Material.

##### Surrogate model inference datasets

To enable fair realism comparisons across surrogates, we generated 100,000 spectra per surrogate model within its native parameter and wavelength domain. Each model was evaluated using its original chromophore absorption data, with extension of wavelength range where possible to better align with our *In Vivo* Human Benchmarking Dataset. Sampling procedures, fitting and training results, and sampled marginal distributions are provided in Secs. S3–S5 and Figs. S2–S3 in the Supplementary Material.

##### Principal component analysis (PCA)

The similarity between simulated and generated spectra and *in vivo* measurements was assessed qualitatively by comparing their projections onto the first two principal components. PCA was fit to image-level, label-averaged spectra from the human dataset to approximate the *in vivo* reflectance manifold. Simulated and generated spectra were then projected into this space and compared visually: overlap with the *in vivo* distribution indicates realism, whereas systematic offsets suggest limitations in the forward model or parameter-sampling strategy.

##### Spectral recall

To quantify realism in a clinically interpretable, tissue class-specific manner, we employed a recall-based metric that measures the proportion of real spectra that have a sufficiently close match among generated spectra. Unlike traditional distributional similarity measures, this approach treats realism primarily as coverage of the real data manifold rather than exact matching of the densities. Spectral recall is both more interpretable in high-dimensional spectral space and more relevant for clinical applications, because *in vivo* datasets are inherently incomplete. Therefore, recall provides a more meaningful measure of physiological utility than similarity metrics.

Our metric builds on established recall concepts from image quality assessment,^49^^,^^50^ which have demonstrated utility for detecting differences in manifold coverage. However, these metrics can be sensitive to distributional shifts, as noted in a recent work on coverage, probabilistic precision, and recall.^51^ Aware of these limitations, we developed a simplified version of neighbor-based recall that has similar pitfalls but is easier to interpret.

Our spectral recall is defined between two datasets of spectra, real spectra $a_{i}\in A$ and simulated or surrogate-generated spectra $b_{j}\in B$, as

$$\text{Recall}(A,B)=\frac{1}{\left|A\right|}\overset{\left|A\right|}{\underset{i=1}{\sum }}\{\begin{array}{ll} 1 & \text{if }\underset{j=1\ldots |B|}{\min}d(a_{i},b_{j})<d_{\max}\\ 0 & \text{otherwise} \end{array}.$$ (5)

The MAE, computed over the $n_{\lambda }$ wavelengths, was selected as the distance metric d

$$\mathrm{MAE}(a_{i},b_{j})=\frac{1}{n_{\lambda }}\overset{n_{\lambda }}{\underset{\lambda =1}{\sum }}|a_{i,\lambda }-b_{j,\lambda }|,$$ (6)

whereas |A| and |B| denote the cardinality of the set of spectra. Our recall metric thus represents the fraction of real spectra that have at least one simulated spectrum within the allowed distance threshold $d_{\max}$.

To determine the maximum allowed distance threshold $d_{\max}$, we evaluated various thresholds, detailed in Fig. S6 in the Supplementary Material. The optimal threshold was selected based on the point of steepest recall increase for each model, with the final threshold chosen as the lowest of these individual optimal thresholds. This approach balanced the risk of setting an overly restrictive threshold against the risk of overestimating real data coverage, resulting in an MAE threshold of $d_{\max}=0.02$. To minimize patient-specific bias and ensure robust statistical analysis, the final recall scores were computed using hierarchical data aggregation: First, the binary spectral recall decision is computed per individual real spectrum, then averaged per patient and class, and finally averaged across patients to obtain tissue-specific recall scores across the entire dataset. This multilevel aggregation strategy ensures that our results are representative of the broader population rather than being dominated by individual patient characteristics.

#### 2.3.3 Clinical use case: *in vivo* ischemia-reperfusion dynamics

We evaluated the clinical potential (RQ3) by assessing whether surrogate-generated spectra can recover organ-specific oxygenation dynamics in a controlled ischemia-reperfusion setting. To maintain full interpretability, we employed a simple nearest-neighbor lookup for $s_{t}O_{2}$ estimation, without additional modeling components.

##### *In vivo* porcine ischemia-reperfusion dataset

To demonstrate the clinical potential of our method, we used an *in vivo* hyperspectral dataset acquired during controlled aortic-clamping experiments in four pigs, also described in Qasim et al.^52^ in the context of a tissue classification study. Each animal was imaged with an HSI system under three perfusion states: physiological baseline, supradiaphragmatic aortic clamping (ischemia), and subsequent reperfusion.

The dataset was acquired with the same TIVITA^®^ Tissue camera system as described in the *In Vivo* Human Benchmarking Dataset. All 152 images were annotated by a clinical expert with pixel-wise semantic tissue labels for the major abdominal structures visible in the field of view, including stomach, small bowel, colon, liver, gallbladder, spleen, peritoneum, subcutaneous fat, visceral fat, muscle, and skin. The hyperspectral images were acquired repeatedly at ∼1-min intervals during ischemia and the reperfusion phase, capturing the temporal evolution of the tissue oxygenation in visceral and peripheral organs.

All procedures were performed in accordance with institutional and national regulations on animal experimentation and were approved by the Committee on Animal Experimentation of the Baden-Württemberg Regional Council in Karlsruhe, Germany (G-161/18, G-262/19). Further information on animal handling and anesthesia protocols can be found in Studier-Fischer et al.^53^

##### Tissue Oxygenation Estimation

For each pixel in the *In Vivo* Porcine Ischemia-Reperfusion Dataset, $s_{t}O_{2}$ was estimated by identifying the single most similar spectrum from our hemoglobin-only Surrogate Model Inference Datasets using MAE. As in the spectral recall analysis, neighbors with MAE>0.02 were discarded to ensure that estimates were only derived from sufficiently close matches in spectral space.

Valid $s_{t}O_{2}$ assignments were then averaged per subject, organ, and time point using the pixel-wise semantic labels, yielding temporal oxygenation trajectories for every subject and organ across baseline, clamping, and reperfusion phases. The recovered physiological trends serve as a qualitative test of the realism and clinical utility of synthetic spectra.

## 3 Results

In line with our three research questions, we evaluated the surrogate model across the three complementary dimensions of performance, realism, and clinical potential.

### 3.1 Surrogate Model Performance

In the following, we report the spectral accuracy, computational speed, and scaling behavior results of the performance experiments in Sec. 2.3.1 of our surrogate model.

#### 3.1.1 Regression fidelity

The surrogate model achieved an MAE of $4.07\times 10^{-5}$ (95% bootstrap CI of the mean $\in [4.06,4.08]\times 10^{-5}$) and a MAPE of 0.0389% (95% bootstrap CI ∈[0.0387,0.0390]%). For comparison, increasing the photon budget tenfold to one billion and simulating identical parameters yielded an MAE of $1.857\times 10^{-5}$ (95% bootstrap CI of the mean $\in [1.854,1.860]\times 10^{-5}$) and a MAPE of 0.02602% (95% bootstrap CI ∈[0.02598,0.02607]%).

**Fig. 4:**
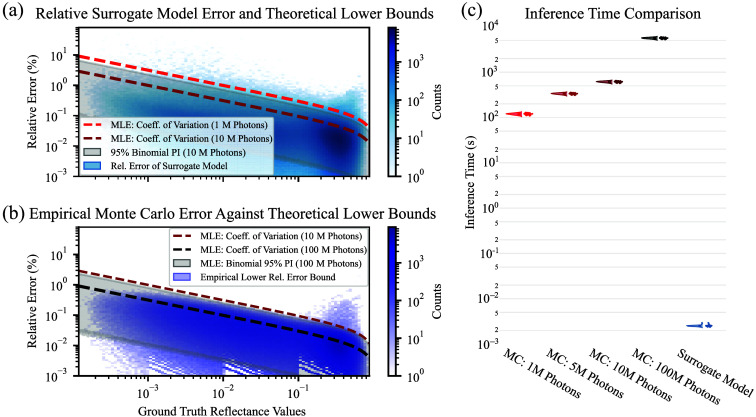
Our surrogate model achieves Monte Carlo-level accuracy with over 100,000× faster inference. (a) The absolute percentage error (APE) of the surrogate model matches the effective theoretical error of Monte Carlo (MC) simulations using 5–10 million photons, with deviations at higher reflectances. (b) Lower error bounds from maximum likelihood estimation (MLE) align with empirical MC error estimates. (c) Inference is 134,000–246,000× faster than MC simulations with comparable accuracy, repeated for 30 independent data batches. Ablation of the inference speed with surrogate model batch size is available in Fig. S1 of the Supplementary Material.

Figure 4(a) presents the APE distribution of the surrogate model, overlaid with theoretical estimates of the CoV and the 95% PIs for photon counts of 10 million, as described in Sec. 2.3.1. Figure 4(b) shows the empirical APE distribution between 100 million and one billion photon simulations, with the theoretical 95% PIs for 100 million photons overlaid for comparison. The CoV for different photon amounts is plotted in both subplots to improve the comparability of the subplots.

The empirical APE in Fig. 4(b) closely aligns with the 95% PI of the theoretical distribution, particularly for lower reflectance values. The surrogate model’s APE falls well within the 10-million-photon PI at lower reflectances, indicating comparable performance in that range. At higher reflectance levels, increased deviation from the expected interval suggests an overall effective equivalence closer to 5 to 10 million photons.

#### 3.1.2 Inference acceleration

Median inference time per batch of 1000 physiological parameter sets with 15 wavelengths was reduced from 336 to 615 s for MC simulations with 5 to 10 million photons (respectively) to 2.5 ms for the surrogate model while maintaining comparable accuracy. This corresponds to a speedup factor between 134,000 and 246,000, as illustrated in Fig. 4(c).

#### 3.1.3 Data efficiency and neural data scaling

**Fig. 5:**
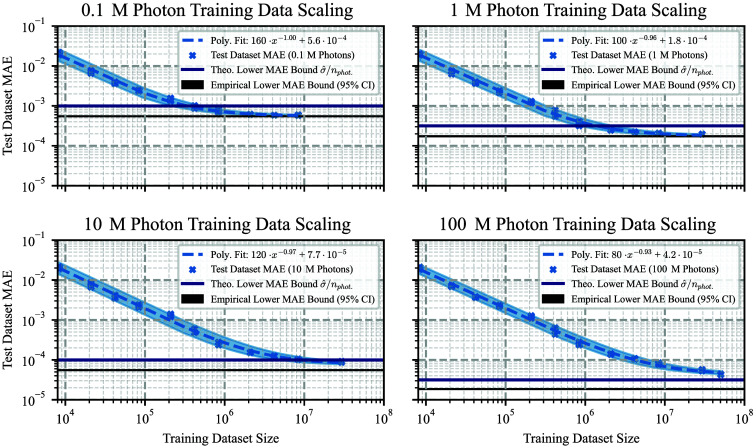
Surrogate model accuracy improves systematically with more training data, approaching the Monte Carlo (MC) error limit. Surrogate models trained on (a) 0.1 million (M)- and (b) 1 M-photon datasets converge on the initial Surrogate Model Development Dataset, whereas the (c) 10 M- and (d) 100 M-photon models have not yet reached their asymptotes, even with extension of the dataset. Performance across all photon levels follows similar power-law scaling, with accuracy differences reflecting photon-dependent MC uncertainty. Results for related works are shown in Fig. S8 in the Supplementary Material.

Figure 5 presents the MAE of surrogate models trained on varying dataset sizes on the test split of our Surrogate Model Development Dataset. The plot includes the fit of the power law from Eq. (4), with standard deviations of the fit parameters shown as uncertainty bands

The surrogate models trained on MC simulations of varying photon counts exhibited clear power-law scaling behavior with respect to training dataset size, converging toward the lower empirical error bound obtained by comparison with the one-billion-photon reference dataset. The asymptotic limit predicted by the maximum likelihood estimator was slightly above this empirical lower bound. Although the overall scaling was similar, the estimated power law exponent b, with standard deviation, ranged from −0.925±0.013 to −1.005±0.018. The model trained on the high-fidelity 100 million photon simulations realized the lowest MAE: The attainable lower error c with standard deviation was $(4.2\pm 1.3)\times 10^{-5}$, and the irreducible loss floor for the current MC simulation photon amount was theoretically estimated to be $\hat{\sigma }=3.15\cdot 10^{-5}$, whereas the 95% bootstrap CI of the empirical lower MAE bound spanned from $[1.85,1.86]\times 10^{-5}$.

### 3.2 Monte Carlo and Surrogate Model Realism

**Fig. 6:**
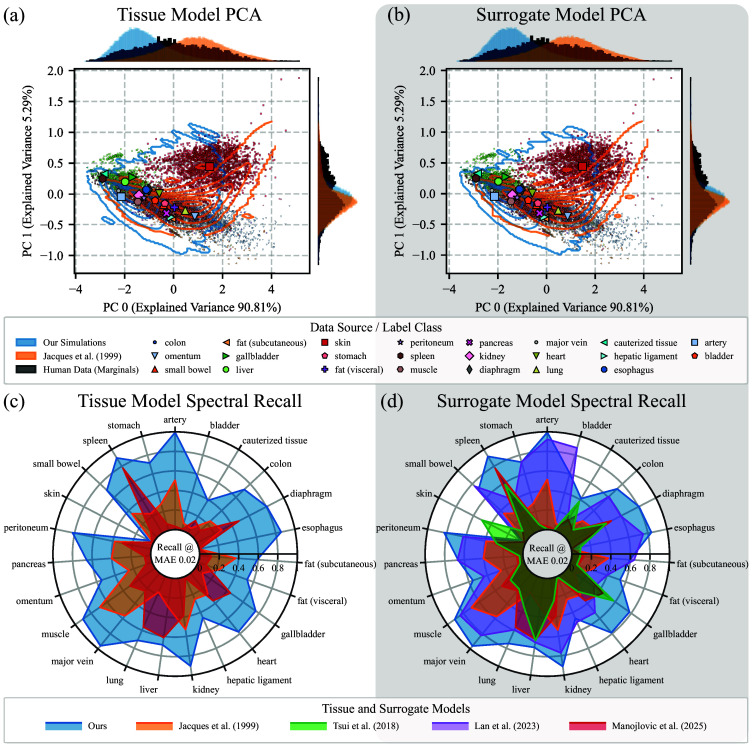
Our surrogate model best captures human tissue spectral diversity. Realism is evaluated for physiological tissue models (left) and their derived surrogates (right). (a), (b) Qualitative: Principal Component Analysis (PCA) embeddings with highlighted class-wise mean principal components show that the best reference model^22^^,^^24^ covers fewer regions of the manifold than ours. Kernel density estimates^54^^,^^55^ overlay the reference model on our implementation. (c), (d) Quantitative: Our model achieves the highest or second-highest recall across all tissue classes. A comparison with the remaining SOTA works, a MAE threshold ablation, and a training dataset size ablation for the recall are included in Secs. S8–S10 of the Supplementary Material.

Figure 6 summarizes the realism analysis of both our physiological tissue and surrogate model, compared against SOTA models. The *in vivo* data exhibit a characteristic topology: All classes except skin form a large, coherent cluster along an axis from spleen and cauterized tissue toward lung and omentum. Both the tissue and surrogate model proposed by Jacques and Bahl et al.^22^^,^^24^ provided the best PCA coverage of the SOTA models and captured some skin and omentum variation that our model misses, but underrepresented the cauterized tissue, spleen, liver, and gallbladder.

Figures 6(c) and 6(d) show spectral recall stratified by tissue class. Our models rank first or second across all tissue classes, increasing mean recall by 41 and 47 percentage points over the MC-simulated physiological tissue models of Jacques^22^ and Manojlovic et al.^13^ and by 13, 42, 47, and 48 percentage points over surrogate baselines from Lan et al.,^30^ Jacques,^22^ Manojlovic et al.,^13^ and Tsui et al.^28^ Among the surrogate models, only Lan et al.^30^ achieves marginally higher recall in five of the 23 tissue classes: bladder, subcutaneous fat, hepatic ligament, pancreas, and small bowel. Skin remains challenging for all methods, including the skin-specific models, with the best, yet limited, coverage achieved by Tsui et al.^28^

### 3.3 Clinical Use Case: *In Vivo* Ischemia-Reperfusion Dynamics

**Fig. 7:**
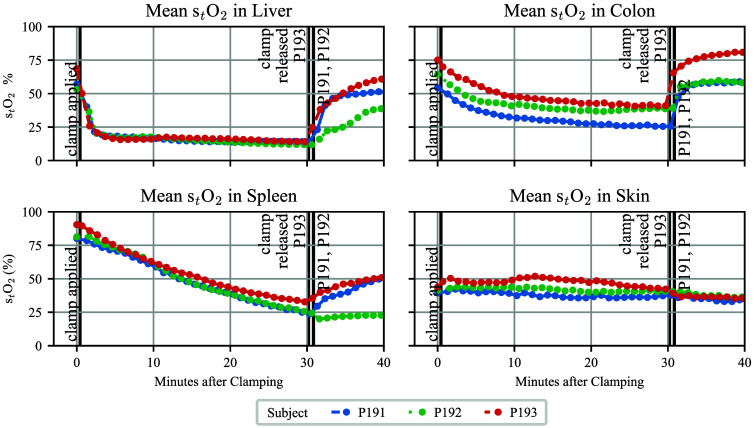
Surrogate-based reflectance spectra estimation captures organ-specific tissue oxygenation ($s_{t}O_{2}$) dynamics during aortic clamping. The mean $s_{t}O_{2}$ trajectories show desaturation and reperfusion in visceral organs, whereas peripheral skin tissue remains unaffected. Detailed, subject-specific plots of all available organs and comparison with Monte Carlo-based $s_{t}O_{2}$ estimates are provided in Fig. S10 of the Supplementary Material.

Figure 7 presents the surrogate-based $s_{t}O_{2}$ trajectories for three pigs undergoing aortic clamping, highlighting characteristic patterns across visceral and peripheral organs: visceral organs consistently decrease in $s_{t}O_{2}$ during clamping, whereas the peripheral skin tissue remains stable across time and subjects.

After clamp application, all visceral organs downstream of the aortic occlusion (colon, liver, spleen) exhibited a drop in $s_{t}O_{2}$. Liver reached the lowest values most rapidly, whereas estimated oxygenation in the colon declined slower and plateaued at slightly higher levels. The spleen showed the slowest and most gradual decline, not reaching a plateau during the 30 min of clamping. Skin maintained values close to its baseline oxygenation value throughout the clamping phase.

At clamp release, all visceral organs displayed an increase in $s_{t}O_{2}$. Colon recovered within less than 5 min, liver showed a more moderate rise over 5 to 10 min, and recovery of the spleen had not finished 10 min after clamp release. Postreperfusion levels differed between subjects, with higher recovery in subjects who also started at higher baseline $s_{t}O_{2}$. Subject P192 showed the smallest and slowest recovery across all visceral organs.

## 4 Discussion

We present a general-purpose surrogate model for multilayer MC simulation of tissue reflectance spectra, designed to achieve high accuracy, scalability, and clinical utility in biomedical optical imaging. To that end, our evaluation followed three complementary objectives: comparing fidelity and efficiency against MC simulations (RQ1), assessing physiological realism on large-scale *in vivo* human data (RQ2), and examining clinical potential in a porcine ischemia-reperfusion experiment (RQ3). The surrogate model matched the accuracy of MC simulations with 5 to 10 million photons while accelerating inference by five orders of magnitude. Our model captured the spectral diversity of 22 out of 23 human tissue classes across more than 140 million human spectra and enabled recovery of organ-specific oxygenation dynamics *in vivo*. Together, these results demonstrate both the fidelity and practical utility of the surrogate model across simulated, observational, and translational settings.

Answering how well our surrogate model matches Monte Carlo accuracy, accelerates inference, and scales with training dataset size, we showed that the surrogate model achieved accuracy comparable to MC simulations with 5 to 10 million photons while accelerating inference 134,000 to 246,000-fold, enabling real-time and large-scale spectral synthesis on consumer GPUs. Our acceleration thus exceeds the speed-ups of 1000 to 40,000× reported for prior neural surrogate models.^28^^,^^30^ Although we compare inference speed and later recall against other SOTA implementations, accuracy and error comparisons are only meaningful when trained on the same dataset and reflectance value distribution. Therefore, cross-paper error comparisons are only provided for completeness in Table S3 in the Supplementary Material.

Training data scaling revealed a close to reciprocal power-law relationship between dataset size and model error, with exponents ranging from −0.925±0.013 to −1.005±0.018. This matches the theoretical expectations of $N^{-1}$ for sufficiently expressive models^56^ and showed convergence toward the empirical lower error bound derived from the one-billion-photon MC reference data. Surrogate models trained using different photon amounts perform similarly before reaching their respective error bounds, allowing to reduce computational cost based on the required surrogate model accuracy by using the minimal required photon amount to generate training data. To our knowledge, this represents the first application of neural scaling analysis in the context of light transport surrogates for tissue optics and provides a principled framework for determining required dataset size and simulation quality given accuracy targets for future dataset design.

The dataset size scaling experiment further showed that retraining the surrogate model on only one million samples (a 50-fold reduction) generated with 10 million photon MC simulations (a 10-fold reduction) still yielded an MAE below $3\times 10^{-4}$. This corresponds to an MAE only an order of magnitude higher than that of the full-dataset model, but at a 500-fold reduced computational cost. These findings imply that a surrogate model of sufficiently high fidelity can also be trained from scratch with substantially reduced training data budgets, depending on the target accuracy.

The PCA and recall analyses assessed how well current physiological MC tissue models and surrogate models captured the variability of *in vivo* human reflectance data. Both approaches based on our multilayer tissue model^7^^,^^16^^,^^36^ achieved consistently strong realism scores, ranking first or second in mean recall across all tissue classes and achieving particularly high coverage for the cauterized tissue, spleen, and gallbladder when compared with the second-best competitor. The close match between the PCA embeddings of simulated and real spectra [Figs. 6(a) and 6(c)] and their recall profiles [Figs. 6(b) and 6(d)] shows that the surrogate not only reaches MC-level accuracy but also preserves the underlying structure of the overall reflectance manifold. Compared with prior models, Lan et al.’s single-layer surrogate^30^ achieved the second-highest recall on average, despite earlier reports suggesting insufficiency of single-layer tissue modeling.^10^^,^^32^ This discrepancy reflects differences in evaluation focus: prior work evaluated reflectance at multiple source-detector separations or in tissue parameter quantification tasks, whereas our validation focused on spectral fidelity and manifold coverage of diffuse reflectance spectra. Across all evaluated simulations and surrogate models, performance trends highlighted a strong influence of chromophore composition and anisotropy in the underlying tissue model on reflectance realism: hemoglobin-based chromophore sets and flexible anisotropy yielded the best PCA overlap and highest recall, whereas skin-specific models^13^^,^^28^ performed comparatively poorly, likely due to their narrow design scope or unrealistically high (millimolar) cytochrome c oxidase concentrations.^57^^,^^58^

Recall values exhibited substantial class-dependent variation and sensitivity to the choice of similarity metrics and threshold. To ensure transparency and reproducibility, we therefore made the threshold selection process explicit. Additional recall ablations showed that spectral realism remained largely unchanged, even when the training set size was reduced more than 2400-fold to match the dataset size of Tsui et al.^28^ This demonstrates that the overall reflectance manifold, as measured by the recall, is preserved across more than three orders of magnitude in training dataset sizes and is largely independent from surrogate model accuracy. These results further emphasize that realism, when defined as recall-based *in vivo* reflectance manifold coverage, is driven primarily by the underlying tissue model design and parameterization rather than by dataset volume alone.

Because the *in vivo* benchmarking dataset predominantly consists of physiologically normal tissue and does not capture the full breadth of pathological variability, we deliberately focused on recall rather than precision. Under these conditions, recall provides a conservative yet meaningful measure of realism: it quantifies how well simulated and generated spectra cover the observed *in vivo* reflectance manifold without assuming that the *in vivo* human dataset is capturing all possible variability. The robust quantitative coverage of 22 out of 23 tissue classes, therefore, confirms the practical utility of the presented surrogate model.

The clinical use case showed that synthetic spectra enabled recovery of realistic organ-specific $s_{t}O_{2}$ trajectories, distinctly separating visceral desaturation from stable peripheral organ responses during aortic clamping. Recovery speeds differed across organs but showed consistent patterns across subjects, despite varying baseline $s_{t}O_{2}$ levels. The speed and magnitude of the oxygenation response depended on blood supply and proximity to the aorta, with the liver showing the strongest response and the spleen the slowest. Intersubject variability was smaller than interorgan differences, resulting in clear organ-specific trajectories, although one subject showed markedly reduced reperfusion responses across all visceral organs. The speed and amplitude of change in $s_{t}O_{2}$ not only align qualitatively with closeness to the clamping site and artery size but also with organ-specific $p_{t}O_{2}$ values reported in the literature: Porcine liver tissue, for example, exhibits lower oxygen tensions than intestinal regions.^59^^,^^60^ Although this qualitative agreement supports the physiological plausibility of the observed $s_{t}O_{2}$ trends, a quantitative relationship between tissue $p_{t}O_{2}$ and optically estimated $s_{t}O_{2}$ can and should not be inferred, given the limited evidence.

Overall, our findings demonstrate that surrogate-generated reflectance spectra support recovery of physiologically meaningful, organ-specific oxygenation dynamics during controlled ischemia and reperfusion, reinforcing the clinical potential of the surrogate for application-level surgical HSI tasks.

Despite its overall strong performance, the proposed surrogate modeling framework has several limitations:

First, the surrogate naturally inherits all assumptions and simplifications of the underlying MC simulations. The surrogate model is constrained to physically plausible optical parameters from established tissue-optics literature and is intended for in-distribution inference rather than extrapolation beyond its training domain. As in many MC tissue models, we used idealized planar geometry, homogeneous optical properties, and a wavelength-independent Henyey-Greenstein phase function. These choices are widely adopted in biomedical optics,^8^^,^^10^^,^^17^^,^^24^^,^^28^^,^^30^^,^^36^ but they limit the ability to capture tissue curvature, spatial heterogeneity, and dynamic perfusion. They also exclude alternative scattering phase functions such as the Mie or Gegenbauer phase function, which may further improve the realism of light-transport modeling, as observed in applications requiring radially resolved reflectance.^61^^–^^63^ Remaining gaps in PCA coverage and recall highlight opportunities to challenge and optimize current modeling assumptions: In particular, chromophore selection and parameter space design may further improve realism within the scope of current multilayer tissue models, as the current scattering amplitude $a_{\mathrm{Mie}}$ and anisotropy g values still differ from some measured *ex vivo* and *in vivo* values.^64^ Importantly, our surrogate model lends itself to further exploration of the current multilayer tissue model paradigm, as its wavelength-independent formulation allows for the direct incorporation of wavelength-dependent scattering amplitude, anisotropy, or refractive index without requiring architectural changes or retraining. This flexibility enables future exploration and integration of more complex tissue models, including alternative chromophore compositions, by simply performing inference on updated tissue model parametrizations.

Second, although the surrogate model greatly reduces inference cost, generating the underlying MC datasets required ∼130 GPU weeks. This corresponds to an estimated energy consumption of four MWh and about 1.5 metric tons of ${\mathrm{CO}}_{2}$-equivalent emissions.^65^ This cost, however, is a one-time investment. The resulting surrogate model is compact, shareable, and widely reusable for inference, given the possibility of device adaptations or domain adaptation frameworks^66^ in postprocessing. Only when our broad parameter space does not cover the new application can the model be retrained. If slightly lower accuracy is acceptable, our data scaling experiment showed that this is feasible with moderate resources of ∼2 GPU weeks and less than 100 kWh. With our surrogate model able to simulate over 100 million single-wavelength reflectances per minute on a single GPU, inference is effectively instantaneous, and the previous data bottleneck now lies in the design of diverse, physiologically plausible training datasets. Future efforts should therefore prioritize targeted exploration of high-impact regions of parameter space for optimal dataset generation.

Third, the clinical use case offers only qualitative insight into ischemia-reperfusion behaviour. Although the estimated $s_{t}O_{2}$ trajectories qualitatively reflect known organ-specific oxygen tensions, such as lower hepatic $p_{t}O_{2}$ compared with intestinal tissue,^60^ the relationship between microvascular oxygen tension and optically estimated $s_{t}O_{2}$ is nonlinear, species-dependent, and strongly influenced by temperature, pH, and $p_{t}{\mathrm{CO}}_{2}$, among other factors.^67^^,^^68^ As a result, quantitative correspondence between $s_{t}O_{2}$ and published $p_{t}O_{2}$ values cannot yet be inferred, underscoring the need for future work beyond phantoms^69^ and skin,^70^ directly linking optical measurements to ground-truth physiological oxygenation.

Finally, limitations arise from the available *in vivo* human dataset used for realism evaluation. Our *in vivo* dataset predominantly represents physiologically normal tissue and therefore underrepresents pathological variability. In this context, recall is the most suitable metric, as it measures coverage of the known spectral manifold without assuming that this manifold is complete, as precision would. Comparisons across studies are further limited by differences in spectral wavelength ranges, which introduce variability in PCA and recall metrics and highlight the broader need for standardized benchmarking conditions.

Looking ahead, expanding datasets to cover application-specific spectral imaging methods, such as dermatological, laparoscopic, and endoscopic HSI, will be essential for clinical translation. Consistent with Setchfield et al.^71^ and Rossberg et al.,^72^ we emphasize the need for standardized large-scale *in vivo* datasets to capture physiological variability and ensure generalization beyond *in vitro* or *ex vivo* validation datasets.

## 5 Conclusion

We introduced a general-purpose surrogate model for multilayer MC simulation of spectral tissue reflectance, achieving MC-level accuracy across a broad optical parameter space, while accelerating inference speeds by five orders of magnitude. By improving realism, efficiency, and scalability, our approach removes a persistent bottleneck in optical tissue simulation and provides a flexible, differentiable model for next-generation biomedical imaging methods. Beyond accelerating spectral simulation, the framework opens opportunities for real-time diagnostics, large-scale inverse problem solving, and compute-efficient, high-throughput pipelines for personalized biomedical optics. Taken together, these contributions establish a scalable platform for realistic spectral data generation and lay the groundwork for more robust optical imaging and AI-driven diagnostic systems.

## Supplementary Material

10.1117/1.JBO.31.2.026004.s01

## Data Availability

The code is available on GitHub, and the generated simulation datasets can be accessed via Zenodo (see GitHub). Human HSI data are not publicly shared due to legal and ethical restrictions. However, access may be granted to qualified researchers on request and subject to institutional approval.
