# Learning to simulate realistic human diffuse reflectance spectra (Erratum)

**DOI:** 10.1117/1.JBO.31.3.039801

**Published:** 2026-03-31

**Authors:** Marco Hübner, Ahmad Bin Qasim, Alexander Studier-Fischer, Maike Rees, Viet Tran Ba, Jan-Hinrich Nölke, Silvia Seidlitz, Jan Sellner, Janne Heinecke, Jule Brandt, Berkin Özdemir, Kris Dreher, Alexander Seitel, Felix Nickel, Caelan Max Haney, Karl-Friedrich Kowalewski, Leonardo Ayala, Lena Maier-Hein

**Affiliations:** a German Cancer Research Center (DKFZ), Division of Intelligent Medical Systems, Heidelberg, Germany; b Heidelberg University, Faculty of Mathematics and Computer Science, Heidelberg, Germany; c Heidelberg University Hospital, National Center for Tumor Diseases (NCT) Heidelberg, a partnership between German Cancer Research Center, Heidelberg, Germany; d Helmholtz Information and Data Science School for Health, Karlsruhe/Heidelberg, Germany; e Heidelberg University, Department of General, Visceral, and Transplantation Surgery, Heidelberg, Germany; f Heidelberg University, Medical Faculty, Heidelberg, Germany; g University Medical Center Mannheim, Medical Faculty of the University of Heidelberg, Department of Urology and Urosurgery, Mannheim, Germany; h German Cancer Research Center (DKFZ), Division of Intelligent Systems and Robotics in Urology (ISRU), Heidelberg, Germany; i DKFZ Hector Cancer Institute at the University Medical Center Mannheim, Mannheim, Germany; j Heidelberg University, Department of Physics and Astronomy, Heidelberg, Germany; k University Medical Center, Hamburg-Eppendorf, Department of General, Visceral, and Thoracic Surgery, Hamburg, Germany; l University Hospital Leipzig, Department of Urology, Leipzig, Germany; m Heidelberg University Hospital, Surgical Clinic, Surgical AI Research Group, Heidelberg, Germany

## Abstract

The erratum list some minor errors in the originally published article, which have since been corrected.

This article [*J. Biomed. Opt.* **31**(2), 026004 (2026) [doi: 10.1117/1.JBO.31.2.026004] was originally published on 26 February 2026 with an error regarding the identification of an enzyme. Specifically, the enzyme referred to as “cytochrome c” should in fact be “cytochrome c oxidase.” To avoid confusion, the two occurrences of “cytochrome c” in the main text and one occurrence in the acknowledgements were corrected accordingly.

In addition, references 58 and 59 were updated by replacing them with the following reference, which correctly pertains to cytochrome c oxidase:

Gemma Bale, Clare E. Elwell, and Ilias Tachtsidis, “From Jöbsis to the present day: a review of clinical near-infrared spectroscopy measurements of cerebral cytochrome-c-oxidase,” *J. Biomed. Opt.* 21(9), 091307 (2016).

These corrections do not affect the results, discussion, or conclusions of the study. In particular, the statement in the discussion — that cytochrome c oxidase concentrations are in the micromolar rather than millimolar range, especially in extracerebral tissue — remains unchanged.

During a review of the published article, the authors identified two additional clarifications:

• In Eq. (4), “−b” has been updated to “b” (typographical sign error in the neural scaling exponent) to correctly represent the inverse relationship, consistent with the reported value of b being close to −1.
• References in the main text to Table S2 (supplemental content) have been revised to refer to Table S3.

The article was corrected and republished 26 March 2026.

