# Supplementary material for: Learning to simulate realistic human diffuse reflectance spectra
Source: J Biomed Opt. 2026 Feb 26;31(2):026004. doi: 10.1117/1.JBO.31.2.026004 (PMC12941064; doi:10.1117/1.JBO.31.2.026004)
Supplement: Supplementary file 1 [file JBO_031_026004_SD001.pdf]

Learning to simulate realistic human diffuse reflectance spectra: Supplementary material

Marco Hübner[1,2,3,*], **Ahmad Bin Qasim**[1,2,3,4], **Alexander Studier-Fischer**[5,7,8,9], **Maike Rees**[1,2], **Viet Tran Ba**[1,2], **Jan-Hinrich Nölke**[1,2], **Silvia Seidlitz**[1,2,3,4], **Jan Sellner**[1,2,3,4], **Janne Heinecke**[1,6], **Jule Brandt**[1,6], **Berkin Özdemir**[5,8,9], **Kris Dreher**[1,10], **Alexander Seitel**[1,3], **Felix Nickel**[4,5,11], **Caelan Max Haney**[8,9,12], **Karl-Friedrich Kowalewski**[7,8,9], **Leonardo Ayala**[1,3,†], **Lena Maier-Hein**[1,2,3,4,6,13,†]

[1]German Cancer Research Center (DKFZ), Division of Intelligent Medical Systems, Heidelberg, Germany
[2]Heidelberg University, Faculty of Mathematics and Computer Science, Heidelberg, Germany
[3]National Center for Tumor Diseases (NCT), NCT Heidelberg, a partnership between DKFZ and University Hospital Heidelberg, Heidelberg, Germany
[4]Helmholtz Information and Data Science School for Health, Karlsruhe/ Heidelberg, Germany
[5]Heidelberg University, Department of General, Visceral, and Transplantation Surgery, Heidelberg, Germany
[6]Heidelberg University, Medical Faculty, Heidelberg, Germany
[7]University Medical Center Mannheim, Medical Faculty of the University of Heidelberg, Department of Urology and Urosurgery, Mannheim, Germany
[8]German Cancer Research Center (DKFZ), Division of Intelligent Systems and Robotics in Urology (ISRU), Heidelberg, Germany
[9]DKFZ Hector Cancer Institute at the University Medical Center Mannheim, Mannheim, Germany
[10]Heidelberg University, Department of Physics and Astronomy, Heidelberg, Germany
[11]University Medical Center, Hamburg-Eppendorf, Department of General, Visceral, and Thoracic Surgery, Hamburg, Germany
[12]University Hospital Leipzig, Department of Urology, Leipzig, Germany
[13]Heidelberg University Hospital, Surgical Clinic, Surgical AI Research Group, Heidelberg, Germany

Keywords: Hyperspectral Imaging, Monte Carlo Simulation, Surrogate Model, Diffuse Reflectance, Tissue Model, Neural Scaling.

*Corresponding Authors. Marco Hübner: marco.huebner@dkfz-heidelberg.de, Lena Maier-Hein: l.maier-hein@dkfz-heidelberg.de
† Shared last authorship

S1 Tissue Model Validation Datasets

Wirkert and Ayala et al.[7,16,36] (Ours) To establish a baseline level of realism by comparing Monte Carlo (MC) simulations with our human benchmarking dataset, we uniformly and independently sampled 500,000 physiological parameter sets across all three tissue layers: $\mathrm{s}_t\mathrm{O}_2 \in [0.001, 1]$, $v_{\text{Hb}} \in [0.001, 0.3]$, $a_{\text{Mie}} \in [5, 50]\text{cm}^{-1}$, $b_{\text{Mie}} \in [0.3, 3]$, $g \in [0.8, 0.95]$, $n \in [1.33, 1.54]$, and $d \in [0.02, 2]$mm. With the fixed bottom layer thickness $d_3 = 20$cm, our tissue model effectively models 14 flexible physical parameters per wavelength (see Tab. 1 in the main text). For each parameter set, we simulated one million photons at 2 nm intervals across the spectral range from 300 to 1000 nm.

To benchmark against existing tissue models, we reimplemented the only two approaches that were based on multi-wavelength physiological parameters and subsampled our simulations to the same common amount of 70,000 MC simulated reflectance spectra.

Jacques and Bahl et al.[22,24] For reimplementing the tissue model by Bahl et al.[24] and fitting the semi-analytical surrogate model of Jacques[22], we simulated 70,000 physiological parameter sets uniformly sampled within the published ranges. Each simulation was run with one million photons across the 300-1000 nm wavelength range at 2 nm intervals, extending the dataset in size, accuracy, and wavelength coverage compared to the original implementation[24].

Manojlovic et al.[13] We simulated 70,000 physiological parameter sets with uniform sampling from the original ranges[13], also using one million photons. To mitigate boundary artifacts, we increased the dermal layer thickness tenfold to 10 cm, reducing photon loss at the lower boundary.

S2 Surrogate Model Development Datasets

Our Development Dataset The dataset used for training our surrogate model consisted of one million sets of physiological parameters obtained by Latin hypercube sampling to efficiently and comprehensively sample the parameter space spanned by our physiological parameters. The dataset was later increased to five million parameter sets to conduct the neural data scaling experiments on a larger dataset. Each simulation used 100 million photons at 15 wavelengths from 300 nm in 46 nm steps, prioritizing parameter diversity over wavelength resolution to provide both a robust and accurate foundation for surrogate model training.

To train the surrogate models proposed in prior work, all related work tissue models were resimulated as faithfully as possible, using original data specifications, photon counts, and parameter range information where available. The physiological datasets of Jacques and Bahl et al.[22,24], and Manojlovic et al.[13] were those detailed in Sec. S1.

Tsui et al.[28] For the reimplementation of Tsui et al.[28], we simulated 30,000 physical parameter sets with 100 million photons each, matching the original work specifications and uniformly sampling from the stated parameter ranges.

Lan et al.[30] The dataset of Lan et al.[30] comprised 5,000 physical parameter sets with 100 million photons each, using Latin hypercube sampling of absorption coefficient μ_a, scattering coefficient μ_s, and anisotropy factor g following the original methodology, with additional constraints $\mu_a \geq 10^{-4}$ and $g \leq 0.9999$ to prevent numerical instabilities.

S3 Surrogate Model Inference Datasets

Our Inference Dataset For our surrogate model, we used our trained surrogate model based on physiological parameters sampled from the Tissue Model Validation Dataset, inferring diffuse reflectance in the range of 300-1000 nm at 2 nm intervals.

Jacques and Bahl et al.[22,24] The semi-analytical surrogate model based on Jacques' formulation[22] was fitted using the corresponding Tissue Model Validation Dataset. For each refractive index, a separate semi-analytical surrogate model was fitted. We selected the surrogate model with the lowest mean absolute percentage error (MAPE) to generate the inference spectra from 300-1000 nm in 2 nm steps, using the same parameter sampling strategy as in the Tissue Model Validation Datasets.

Tsui et al.[28] Physiological parameters were uniformly sampled within the model inference bounds specified in the original publication[28]. Rejection sampling was applied to discard samples falling outside the model's original physical parameter training domain. The wavelength range was restricted to 460-760 nm in 2 nm steps due to the collagen absorption spectrum specified in the original work[28].

Lan et al.[30] To match the in vitro validation conditions of liquid phantoms with dissolved hemoglobin powder utilized in the original work[30], we constructed a compatible physiological model using hemoglobin as the sole absorber, with parameters uniformly sampled from: $\mathrm{s}_t\mathrm{O}_2 \in [0.001, 1.0]$, $v_{\mathrm{Hb}} \in [0.001, 1.0]$, $a_{\mathrm{Mie}} \in [2.5, 60]\mathrm{cm}^{-1}$, $b_{\mathrm{Mie}} \in [0.1, 4]$, and $g \in [0.8, 0.9999]$. Rejection sampling was employed to exclude samples falling outside the surrogate model's training space until the 100,000 valid spectra were obtained, in the range from 450-650 nm at 2 nm intervals.

Manojlovic et al.[13] We employed the same physiological parameter sampling procedure as in the corresponding Tissue Model Validation Dataset to generate the inference dataset.

All surrogate model-generated inference datasets comprised 100,000 reflectance spectra to avoid dataset size effects in the evaluations. Detailed information on fitting performance metrics and sampled marginal distributions for comprehensive analysis and reproducibility are provided in the following three sections Sec. S4-S7.

S4 Parameter and Wavelength Ranges of Related Work Tissue Simulations

To contextualize the scope and flexibility of our proposed dataset, Tab. S1 compares the physical parameter and wavelength ranges used in prior tissue reflectance simulation studies. These ranges reflect each study's assumptions and constraints, including target applications, available optical property data, and hardware limitations. Our dataset extends this landscape by covering a broader physiological range, including cases of very high absorption and scattering, enabling more robust and broader applicable surrogate modeling. Deviations from original implementations are highlighted in bold, indicating where adjustments for consistent benchmarking were made.

Table S1 Broader parameter coverage has the potential to improve tissue models' applicability across diverse clinical settings. Comparison of physical parameter and wavelength ranges used in tissue reflectance simulations across different studies. The parameter intervals [min, max] reflect each study's design choices, shaped by intended application, available chromophore data, and hardware constraints. Our proposed dataset offers the broadest parameter coverage, including cases with both very large and very small absorption and scattering. Deviations from original implementations are highlighted in **bold.**

Reference Work	μ_a[cm^{-1}]		μ_s[cm^{-1}]		g		n		d [cm]		Wavelength Range [nm]	
	min	max	min	max	min	max	min	max	min	max	min	max
Jacques and Bahl et al.[22,24]	6.8 $\times 10^{-3}$	188	9.4	1968	0.7	0.9	1.33, 1.35, and 1.44		3 (fixed)		**300**	**1000**
Tsui et al.[28]	0.012-350 layer dep.		10-1000 layer dep.		layer dep., fixed		layer dep., fixed		layer dep., variable		**460**	760
Lan et al.[30]	$\mathbf{10^{-4}}$	10	100	350	0.8	**0.9999**	1.35 (fixed)		20 (fixed)		450	650
Manojlovic et al.[13]	0.31	196	75	504	wavelength dep., fixed		wavelength dep., fixed		0.01 (epidermis), **10** (dermis)		**300**	**1000**
Ours	1.7 $\times 10^{-3}$	804	3.8	4540	0.8	0.95	1.33	1.54	0.002	0.2	300	1000

Table S2 The fitted parameters $M1$-$M3$ of the semi-analytical homogeneous tissue model by Jacques[22] are similar to those reported by Bahl et al.[24] but not consistent within the standard deviations of the fitting parameters (not reported). These discrepancies indicate differences in dataset generation, wavelength range of the fit, and implementation between our reimplementation and theirs.

	$n = 1.33$		$n = 1.35$		$n = 1.44$	
	Ours	Bahl et al.[24]	Ours	Bahl et al.[24]	Ours	Bahl et al.[24]
M1	7.2559	7.0188	7.3239	7.1185	7.4669	7.0438
M2	0.0718	0.2464	0.1070	0.2750	0.3829	0.6902
M3	2.0784	4.2241	2.2614	4.2571	3.0265	4.1449

S5 Surrogate Model Fitting and Training Performance

To ensure a fair and reproducible comparison with existing surrogate models, we reimplemented several reference models following their published specifications. This section presents supplementary validation results supporting our main text. Tab. S2 compares key fitted parameters of the semi-analytical tissue model originally proposed by Jacques[22] and later reimplemented by Bahl et al.[24], highlighting subtle discrepancies attributable to the different wavelength ranges in consideration. Tab. S3 states the minimum test errors reported by the original surrogate model works, alongside the test errors from our reimplementation under matched conditions. These results validate the consistency of our reimplementation while underscoring differences in model performance across datasets. Metrics are defined as follows:

$$\text{Absolute Percentage Error (APE)}(y, \hat{y}) = 100 \cdot \frac{|\hat{y} - y|}{y}$$

$$\text{Mean Absolute Percentage Error (MAPE)}(Y, \hat{Y}) = \frac{1}{|Y|} \sum_{i=1}^{|Y|} \text{APE}(Y_i, \hat{Y}_i)$$

$$\text{Normalized Absolute Error (NAE)}(y, \hat{y}) = \frac{|\hat{y} - y|}{y}$$

$$\text{Normalized Mean Absolute Error (NMAE)}(Y, \hat{Y}) = \frac{1}{|Y|} \sum_{i=1}^{|Y|} \text{NAE}(Y_i, \hat{Y}_i)$$

$$\text{Normalized Root Mean Square Error (NRMSE)}(Y, \hat{Y}) = \sqrt{\frac{\sum_{i=1}^{|Y|} (\hat{Y}_i - Y_i)^2}{\sum_{i=1}^{|Y|} Y_i^2}}$$

Table S3 Our reimplementation of state-of-the-art (SOTA) surrogate models achieves lower test error than originally reported across all comparable cases. For each surrogate model, we report the **minimum test error from the original publication in boldface**, including uncertainty where available, alongside the test error from our reimplementation under matched conditions. The metrics used are mean absolute percentage error (MAPE), absolute percentage error (APE), normalized mean absolute error (NMAE), normalized absolute error (NAE), and normalized root mean square error (NRMSE). To capture the variability in model error, we include the central 95% percentile of the respective metric values where possible. Note that error values indicate each model's ability to fit its own reference dataset and are not directly comparable across datasets.

Reference Work	MAPE [%]	APE [%] 95% Perc.		NMAE	NAE 95% Perc.		NRMSE	Comments
		min	max		min	max		
Jacques and Bahl et al.[22,24]	7.55	0.023	76	0.08	2.3×10^{-4}	0.76	**0.030 (±0.048) (reported), 0.0089 (reimplemented)**	We report and use the model with $n = 1.44$ due to best MAPE fit.
Tsui et al.[28]	**1.28 (±1.19) (reported), 0.39 (reimplemented)**	0.011	1.6	0.004	1.1×10^{-4}	0.016	0.0039	Varies with source-detector separation (SDS).
Lan et al.[30]	0.35	0.0032	1.9	**0.018 (reported), 0.0034 (reimplemented)**	0.3×10^{-4}	0.019	0.0017	Varies with source-detector separation (SDS).
Manojlovic et al.[13]	0.18	0.006	0.6	0.0018	0.6×10^{-4}	0.006	0.0017	No surrogate model error reported.

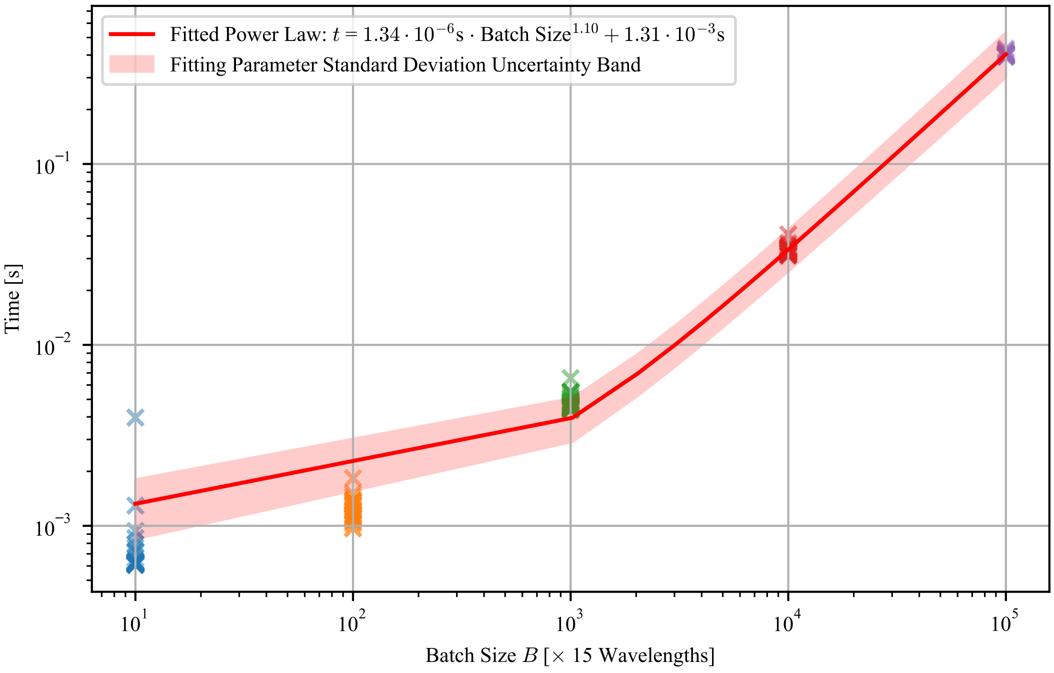

Fig S1 Inference throughput scales with batch size following a simple power law, highlighting efficiency gains for large batches despite fixed preprocessing and GPU transfer overhead. Batch sizes exceeding 1,000 samples (each with 15 wavelengths) are required for the surrogate model to achieve over 100 million single-wavelength inferences per minute due to a fixed preprocessing and GPU transfer overhead of approximately 1 ms.

S6 Ablation of Surrogate Model Inference Speed with Batch Size

To better understand the runtime behavior of our surrogate model and identify practical limits for high-throughput simulation, we conducted an ablation study of inference speed as a function of batch size. While surrogate models offer orders-of-magnitude faster inference compared to Monte Carlo (MC) simulations, their performance is limited by (fixed) overhead costs such as data preprocessing and GPU memory transfer. By systematically increasing the number of physiological samples per batch, we characterized how throughput scales and determined the batch sizes required to saturate GPU utilization and surpass critical throughput thresholds.

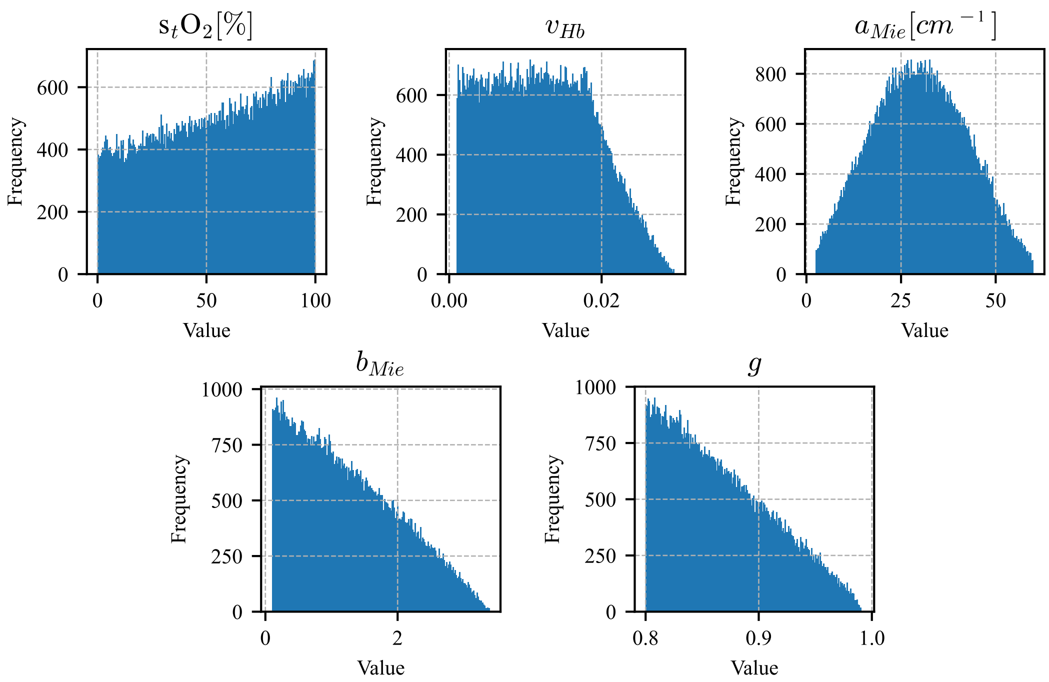

Fig S2 Dataset of physiological parameters used to generate the inference dataset with the reimplemented surrogate model of Lan et al.[30], ensuring physical in-distributionness. Parameters were uniformly sampled from the following ranges: $\mathrm{s_tO_2} \in [0.001, 1]$, $v_{\mathrm{Hb}} \in [0.001, 1]$, $a_{\mathrm{Mie}} \in [2.5, 60]\mathrm{cm}^{-1}$, $b_{\mathrm{Mie}} \in [0.1, 4]$, and $g \in [0.8, 0.9999]$. Sampled physiological parameter sets with transformed physical parameters falling outside the surrogate model's training range were rejected. Sampling continued until 100,000 valid physiological samples were obtained, and reflectance spectra were subsequently generated using the trained surrogate model.

S7 Inference Dataset Parameter Marginals

To enable consistent and fair evaluation of surrogate model performance across reimplemented methods, we generated inference datasets tailored to the original input parameter ranges of each model. Figure S2 and S3 show the distributions of physiological parameters sampled for the Lan et al.[30] and Tsui et al.[28] models, respectively. Sampling was performed uniformly within the specified bounds, with any samples producing physical parameters outside the surrogate's training domain discarded, yielding the presented inference parameter distributions. This procedure ensured that each model was evaluated on physiologically valid, in-distribution inputs, allowing a controlled comparison of spectral predictions.

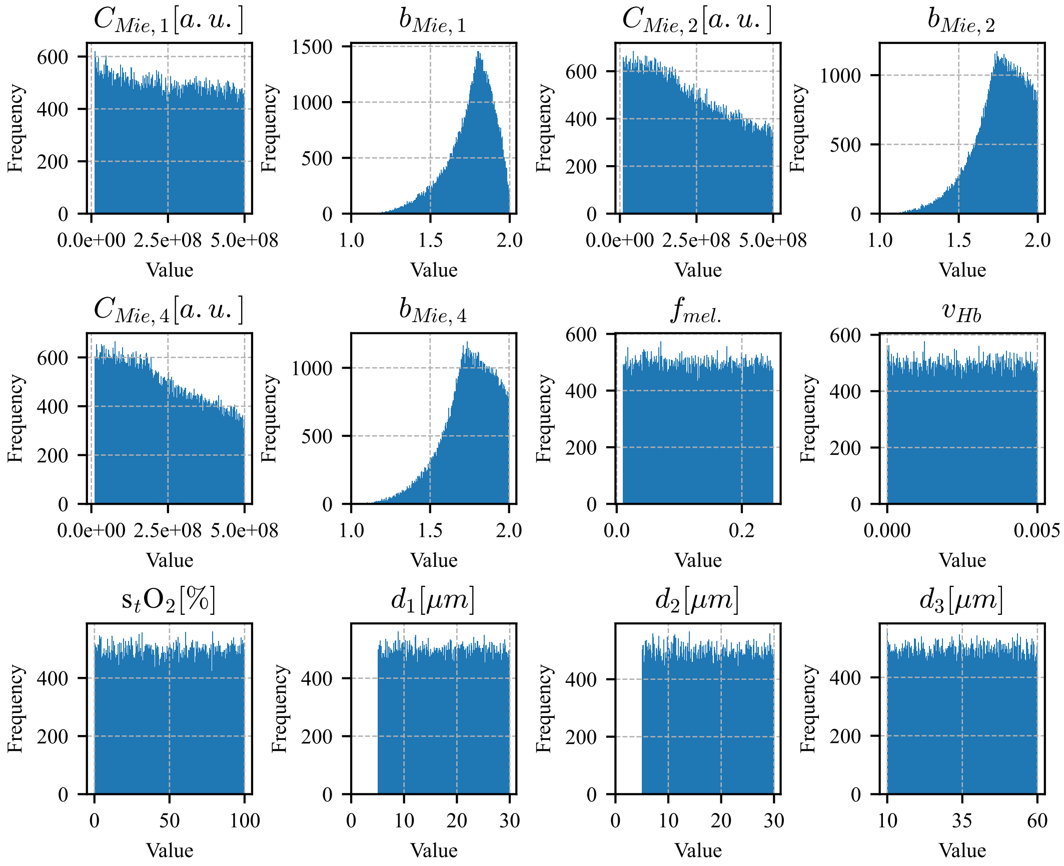

Fig S3 Dataset of physiological parameters used to generate the inference dataset with the reimplemented surrogate model of Tsui et al.[28], ensuring physical in-distributionness. All parameters were uniformly sampled from the ranges reported in the original publication[28]. Sampled physiological parameter sets with transformed physical parameters falling outside the surrogate model's training range were rejected. Sampling continued until 100,000 valid physiological samples (i.e., spectra) were obtained, and reflectance spectra were subsequently generated using the trained surrogate model.

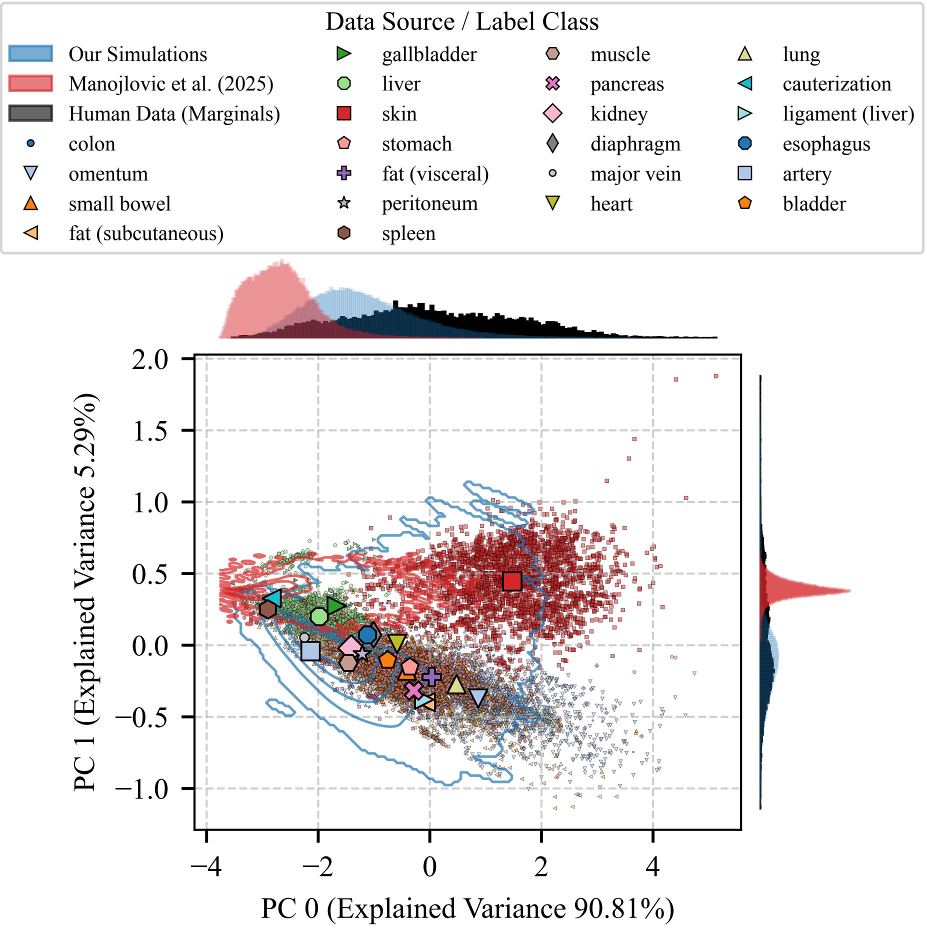

Fig S4 The surrogate model of Manojlovic et al.[13] provides less coverage across tissue classes than our tissue model and the tissue model of Jacques[22] and Bahl et al.[24] shown in the main text. This qualitative comparison complements Fig. 5a. The Manojlovic et al.[13] model exhibits less overlap with in vivo reflectances, covers fewer tissue classes, and shows greater deviation from and class-specific mean principal components.

S8 Additional Tissue and Surrogate Model PCA Analysis

To complement Fig. 5 in the main text, which shows Principal Component Analysis (PCA) comparisons with the best-performing tissue and surrogate models, we include here the remaining PCA visualizations for all related work. These additional plots provide a comprehensive overview of the spectral distributions generated by each surrogate model in comparison to real in vivo data and our model, using kernel density estimation (KDE)[52,55] to overlay the simulated and generated data onto the in vivo data points. Models are presented chronologically and differ in the number of tissue classes covered.

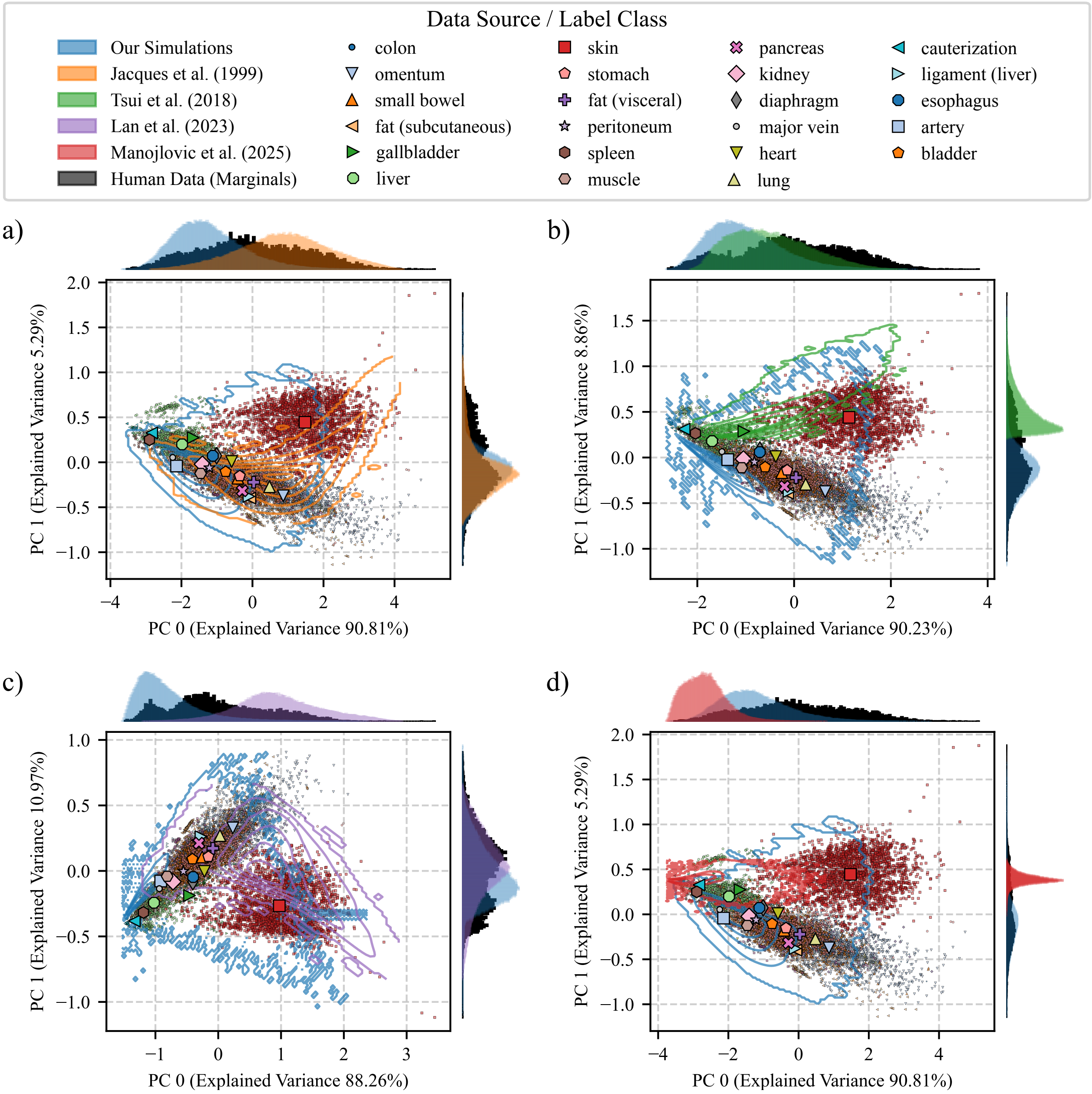

Fig S5 Principal Component Analysis (PCA) shows that our surrogate model achieves the best spectral coverage of in vivo data across all surrogate models. This qualitative comparison complements Fig. 5b and compares all reimplemented surrogate models with our surrogate model and in vivo data in PCA space. To allow comparison, PCA was computed over the shared wavelength ranges between real and surrogate data: 500-1000 nm for Jacques and Bahl et al.[22,24] and Manojlovic et al.[13], 500-760 nm for Tsui et al.[28], and 500-650 nm for Lan et al.[30], based on each model's constraints and our camera's range. Kernel density estimates (KDE) were computed using consistent parameters[52,55]. Models are shown chronologically. Jacques and Bahl et al.[22,24] cover most tissue classes, Lan et al.[30] some, and Tsui et al.[28] and Manojlovic et al.[13] only a few.

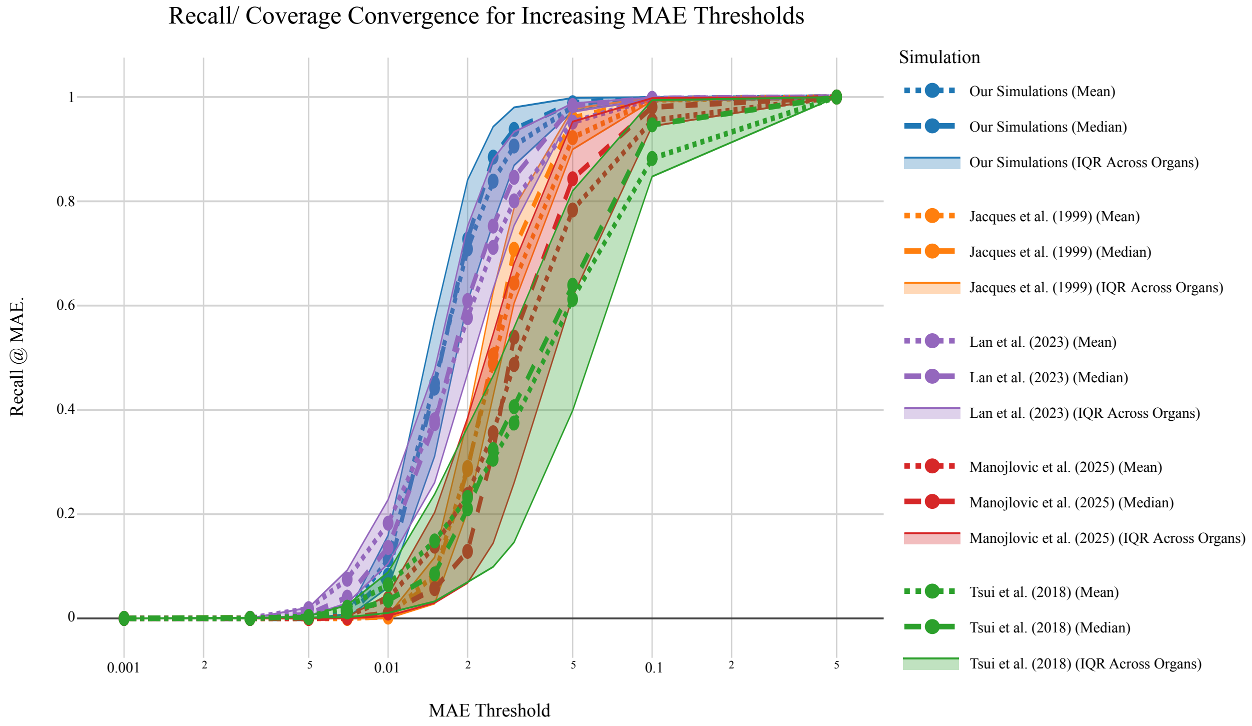

Fig S6 Selection of the optimal mean absolute error (MAE) threshold (0.02) for spectral recall balances minimizing MAE and maximizing recall sensitivity across surrogate models. Multiple thresholds were evaluated to identify the point where the spectral recall increases most while maintaining low MAE, ensuring a robust and interpretable realism metric.

S9 Ablation of the MAE Threshold for Inclusion in the Recall

To ensure robust and fair spectral recall evaluation across surrogate models, the selection of the mean absolute error (MAE) threshold used for recall inclusion is made transparent in Fig. S6 by showing alternative outcomes for multiple threshold values. The threshold directly influences recall sensitivity and specificity, and must avoid both underestimation and oversaturation of performance. Therefore, the threshold was selected corresponding to the earliest and steepest increase in recall across models, yielding an MAE threshold of 0.02.

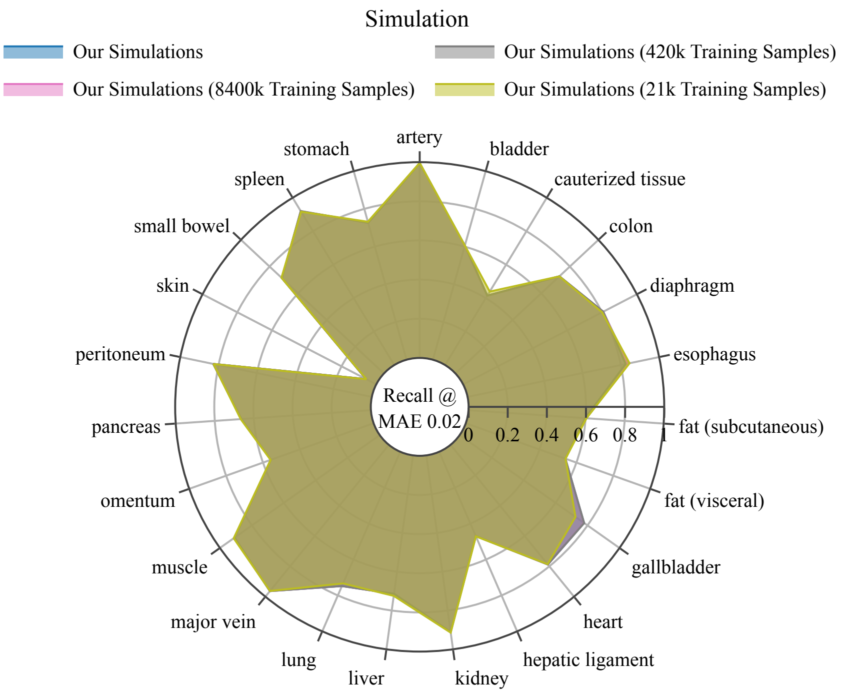

Fig S7 Recall remains stable across training dataset sizes ranging from 50.4 million to 21,000 samples (plots overlap), demonstrating that model realism is retained even with drastically reduced training data. The smallest dataset (21,000 samples) corresponds to the training size used by Tsui et al.[28]. This experiment confirms that the surrogate model preserves sufficient realism as defined by recall (at mean absolute error (MAE) < 0.02) when trained on dataset volumes comparable to prior work.

S10 Ablation of Recall with Training Dataset Size

To investigate the dependence of our surrogate model's realism on training data volume, we conducted an ablation study computing the recall for surrogate models trained on different training dataset sizes. Specifically, we compared models trained on progressively reduced subsets of our dataset containing 50.4 million, 8.4 million, 420,000, and 21,000 physical parameter-reflectance pairs. The latter matches the training dataset size used by Tsui et al.[28], enabling a direct comparison to previous surrogate-based implementations.

As shown in Fig. S7, the surrogate model maintains high spectral realism even when trained with substantially fewer samples. Across all labels, the recall at an MAE < 0.02 remains nearly constant down to 21,000 samples, highlighting robust in vivo data coverage, even when the training dataset size is decreased 2,400-fold. This observation suggests that a sufficiently capable model architecture, the training procedure, and physically grounded dataset design, rather than sheer data volume, drive the improvements in realism and applicability across tissue classes. Consequently, even small-scale training regimes can achieve realism comparable to the full-scale model, supporting the computational feasibility of the proposed surrogate modeling approach.

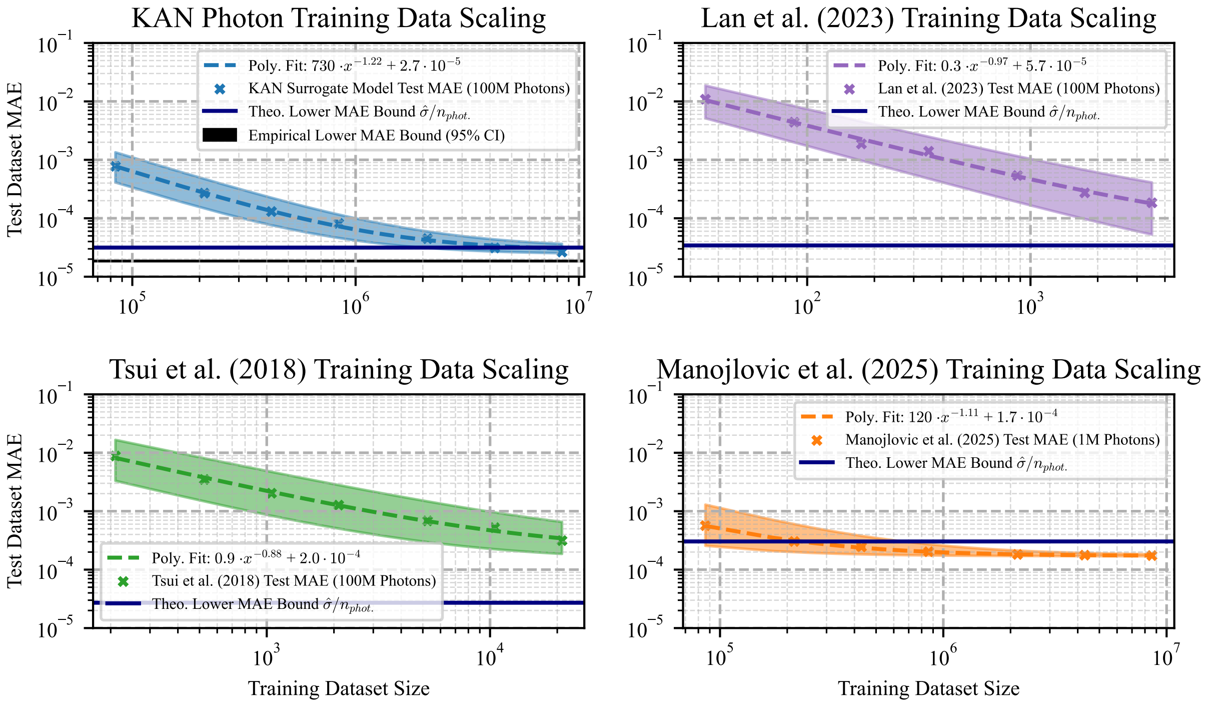

Fig S8 Dataset scaling shows a faster convergence of Kolmogorov-Arnold Networks (KANs) compared to the simpler fully connected architecture and varying states of convergence for the datasets used in related work. We analyze dataset scaling of KANs[77] on our data and perform ablation studies on related surrogate models using varied dataset fractions (1%-100%) for training. While KANs converge faster, they exhibited poorer worst-case robustness, leading to our choice of a simpler fully connected architecture. Among the related work surrogate models, Tsui et al.[28] and Lan et al.'s[30] models, trained on small datasets, have not converged to the estimated lower error bound, whereas Manojlovic et al.'s[13] model surpasses it. As shown in the main paper, empirical lower bounds were consistently lower than the theoretical estimate $\hat{\sigma}$. Although we did not empirically verify the lower bound using higher-photon Monte Carlo simulations, the observed results of Manojlovic et al.[13] are thus expected to be consistent with our empirical lower error bound estimate.

S11 Ablation of Neural Data Scaling Behaviour

This section investigates how surrogate model performance scales when using alternative model architectures and baseline surrogate models from related work. Additionally, we assessed the behavior of the empirical MC lower error bound with varying training dataset photon amounts.

Figure S8 extends the analysis to alternative neural architectures and surrogate models from related work. Kolmogorov-Arnold Networks (KANs)[77] and baseline models were evaluated by training on progressively larger subsets of their respective datasets. While KANs converged using less than 20% of our full training dataset, they showed poorer worst-case performance, supporting our choice of a simpler fully connected architecture. Related work surrogate models did not always reach the estimated lower error bound, emphasizing the importance of sufficient training data volume.

Figure S9 demonstrates that the empirical MC error decreases with a power law exponent with standard deviation $b_\gamma = 0.5027 \pm 0.0013$ (of the power law: $\text{Loss}(N_{\text{photons}}) = a \cdot N_{\text{photons}}^{-b_\gamma} + c_\gamma$) as the photon count increases, aligning with naive expectation $b_\gamma \sim 0.5$ for MC simulations, based on

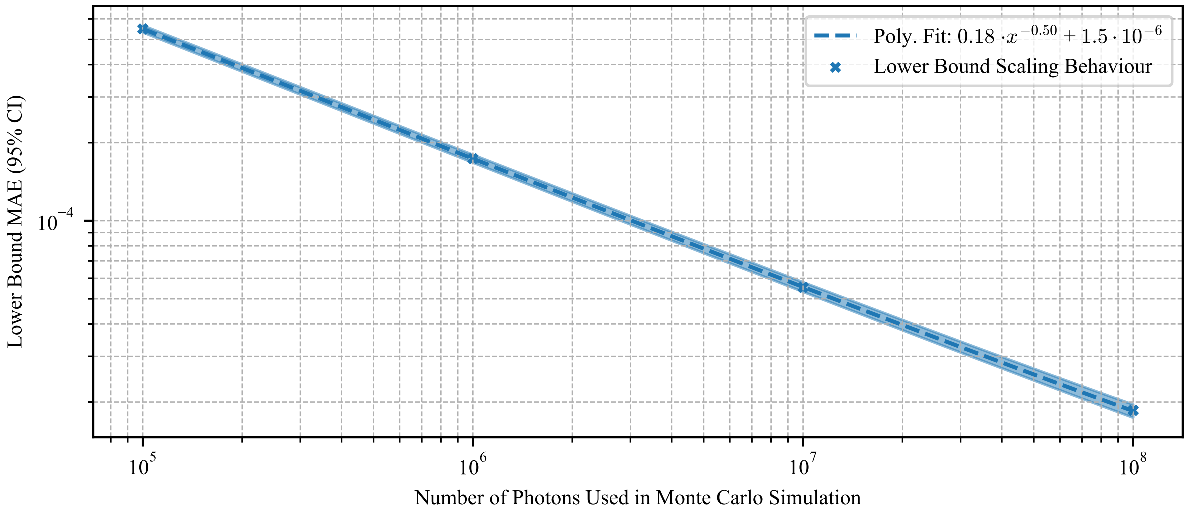

Fig S9 Empirical Monte Carlo (MC) error decreases predictably with photon count, validating the theoretically expected power-law scaling of MC accuracy. The empirical MC lower bound error follows a consistent power-law scaling with an exponent (and standard deviation) of -0.5044 ± 0.0018 across photon counts, closely matching the theoretical expectation that the standard error of MC simulations decreases proportionally to $1/\sqrt{n}$ for sample size n.

the law of large numbers. This supports the use of our empirical MC error estimates as a reliable lower error bound for evaluating surrogate model performance.

S12 Subject-Specific s_tO_2 Dynamics Under Aortic Clamping: Additional Data

To provide a complete overview of all available organ measurements, including more peripheral tissues, we display the remaining available data in this section. Furthermore, we compared surrogate-generated s_tO_2 trajectories with those obtained from MC-based estimation across all visceral and peripheral organs for each pig.

Figure S10 shows that both surrogate and MC-based estimation result in almost indistinguishable temporal patterns. Visceral organs downstream of the aortic occlusion (colon, small bowel, liver, spleen, stomach, gallbladder, visceral fat) exhibited the same rapid declines in s_tO_2 immediately after clamping as in the main text. The liver showed the steepest and earliest drop, colon and small bowel followed similar pronounced trajectories at slightly higher levels, while stomach, visceral fat, and gallbladder showed comparable but slightly more gradual declines with higher plateaus, whereas the spleen displayed the most gradual decrease. All peripheral tissues (skin, subcutaneous fat, peritoneum, muscle) remained comparatively stable throughout the clamping phase, showing only minor deviations from their baseline oxygenation.

After clamp release, most visceral organs showed a marked s_tO_2 increase. Colon, small bowel, stomach, and visceral fat recover within the first few minutes, liver exhibited a slower rise, and spleen remained in a prolonged recovery phase during the 10-minute reperfusion window. As in the main text, the time of recovery varied across organs but was consistent across subjects. Small bowel and colon responded similarly due to their shared blood supply, whereas liver, spleen, stomach, and visceral fat showed distinct organ-specific response speeds and magnitudes. In contrast, the gallbladder did not display a clear reperfusion-related increase and instead showed a rapid drop. The cause of this decline remains uncertain, as it is based on only few annotated pixels, occurred only in the subject with generally poor visceral recovery, and may be influenced by the presence of bilirubin, a chromophore not represented in our tissue model. The overall reperfusion patterns were consistently reproduced within each organ class, while subject-specific differences in baseline s_tO_2 and recovery amplitude remained. Additionally, subject P192 displayed reduced recovery across all visceral organs. Across organs and subjects, surrogate-based estimates overlaped with the MC-based reference curves (circles and diamonds are hardly distinguishable), indicating that the surrogate model provided sufficiently similar spectra to the MC reference data, and thus enables comparable recovery of physiologically meaningful, organ-specific oxygenation dynamics during ischemia and reperfusion.

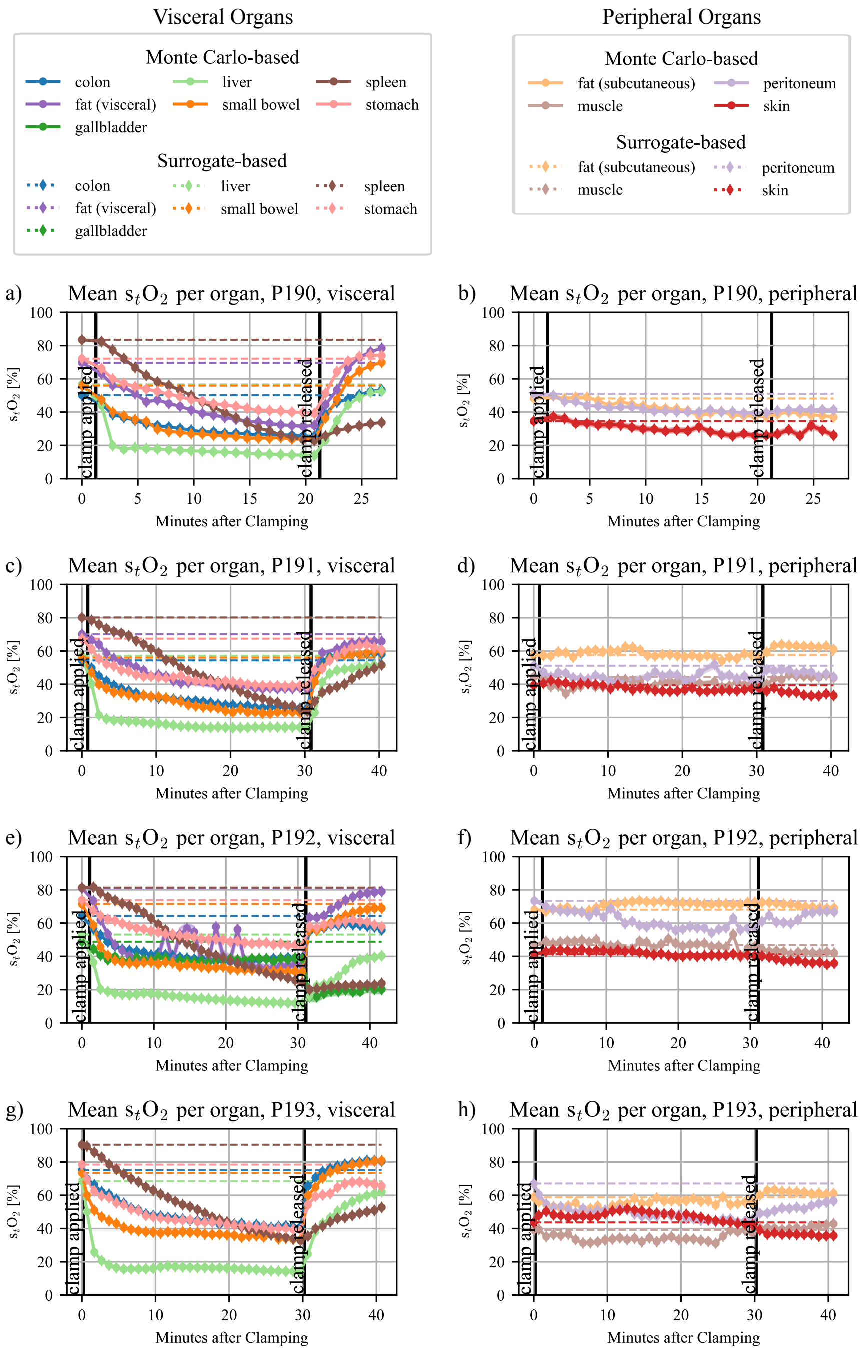

Fig S10 Subject-specific organ-level s_tO_2 trajectories Monte Carlo (MC) and surrogate-based estimation are nearly identical. Panels (a–h) show mean tissue oxygen saturation (s_tO_2) per organ over time for each pig (P190-P193), comparing surrogate-based nearest-neighbor assignment (solid lines and circles) with Monte Carlo-based assignment (dashed lines and diamonds). Across organs and subjects, surrogate-based s_tO_2 trajectories closely match the corresponding MC-based reference curves, preserving both the qualitative organ patterns and the subject-specific baseline differences. Clamp application and release times are indicated by vertical black lines.

S13 Shapley Values Validate Physical Sensibility of the Surrogate Model

To better understand the influence of the inputs to our surrogate model on the reflectance output, we computed SHAP (SHapley Additive exPlanations) values for all input features[78]. SHAP provides a principled way to assess the relative importance of each parameter by quantifying its contribution to the model's output. This analysis offers statistically grounded insights into how different tissue properties influence the predicted spectral reflectance and allows to evaluate whether the model's behavior aligns with physical intuition.

High anisotropy, which implies more forward scattering, lowers diffuse reflectance, as expected. A higher top-layer refractive index also reduces reflectance. This may appear counterintuitive, but it aligns with the fact that the model predicts only diffuse reflectance: Increased refractive index mismatch leads to more specular reflection, which is not captured in the MC model output and thus results in reduced predicted diffuse reflectance. Specular reflection components were excluded from the modeling process, as they can be added post hoc using the Fresnel equations. Interestingly, input features that have often been kept fixed in previously proposed tissue models, such as top-layer anisotropy and refractive index, have a similar or even more influence on the prediction than third-layer absorption. Together with the high recall of Lan et al.[30], this suggests that the flexibility of anisotropy may be more influential than layer number in representing tissue inhomogeneity.

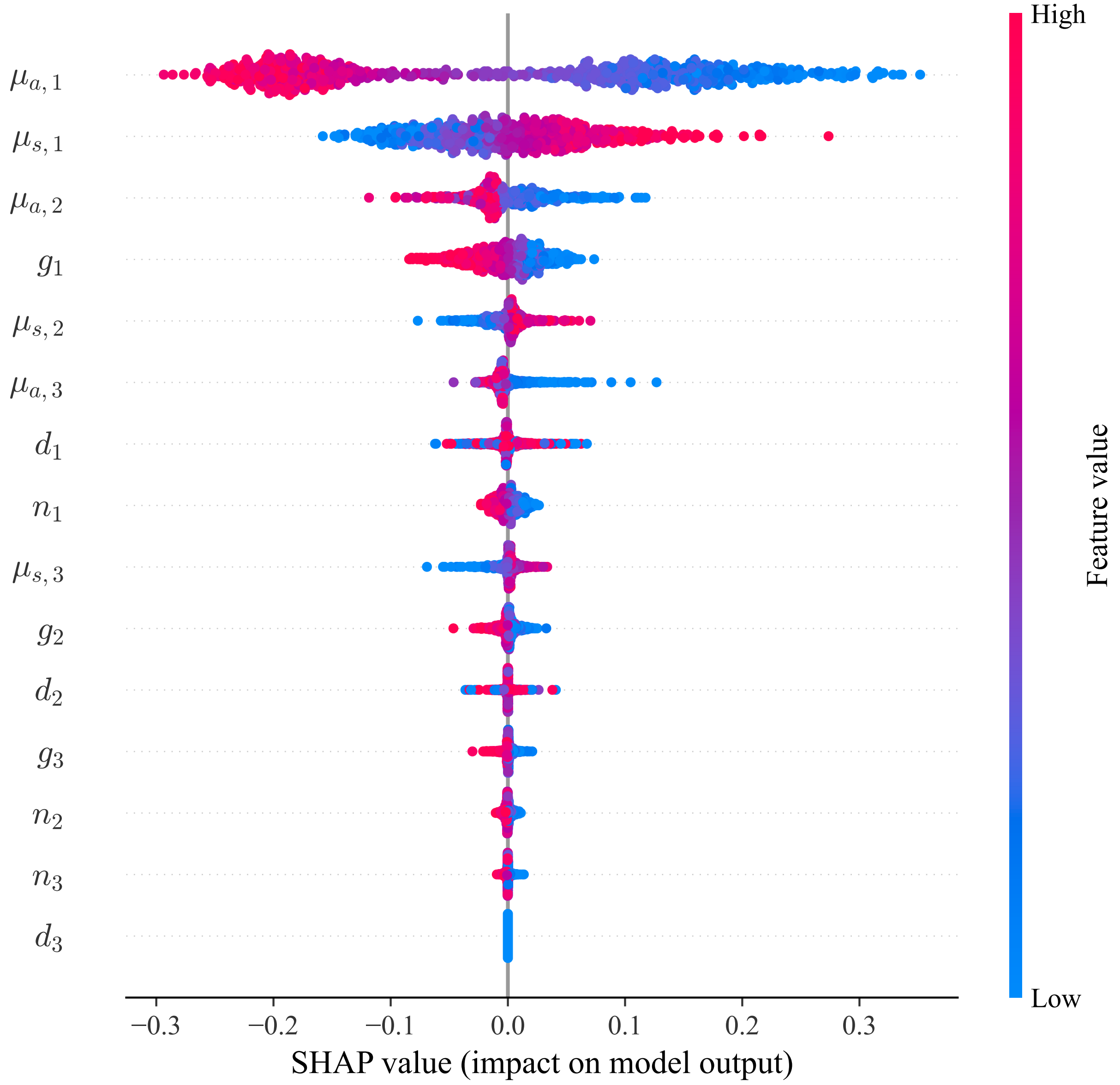

Fig S11 SHapley Additive exPlanations (SHAP) analysis indicates that top-layer tissue parameters predominantly drive surrogate model reflectance predictions, highlighting key physical influences. Absorption and scattering coefficients of the top tissue layer have the strongest impact, where higher absorption decreases reflectance and increased scattering raises it, aligning with physical expectations. Deeper layers, particularly the third, contribute only marginally to the model's output.
